# Mechanism for Peptide Bond Solvolysis in 98% w/w Concentrated Sulfuric Acid

**DOI:** 10.1021/acsomega.4c10873

**Published:** 2025-01-22

**Authors:** Janusz J. Petkowski, Maxwell D. Seager, William Bains, John H. Grimes, Sara Seager

**Affiliations:** † Faculty of Environmental Engineering, Wroclaw University of Science and Technology, 50-370Wroclaw,Poland; ‡ JJ Scientific, Mazowieckie, Warsaw 02-792, Poland; § Department of Chemistry and Biochemistry, Worcester Polytechnic Institute, Worcester, Massachusetts 01609, United States; ∥ Nanoplanet Consulting, Concord, Massachusetts 01742, United States; ⊥ School of Physics & Astronomy, Cardiff University, 4 The Parade, Cardiff CF24 3AA, U.K.; # Rufus Scientific, Melbourn, Herts SG8 6ED, U.K.; ∇ Complex Carbohydrate Research Center, University of Georgia, 315 Riverbend Road, Athens, Georgia 30602, United States; ○ Department of Earth, Atmospheric and Planetary Sciences, Massachusetts Institute of Technology, 77 Massachusetts Avenue, Cambridge, Massachusetts 02139, United States; ◆ Department of Physics, Massachusetts Institute of Technology, 77 Massachusetts Avenue, Cambridge, Massachusetts 02139, United States; ¶ Department of Aeronautics and Astronautics, Massachusetts Institute of Technology, 77 Massachusetts Avenue, Cambridge, Massachusetts 02139, United States

## Abstract

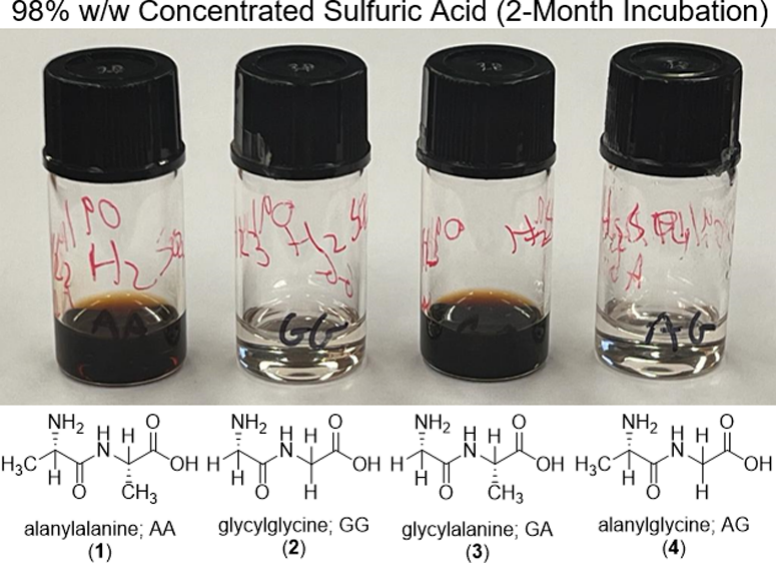

We propose a mechanism for the solvolysis of the peptide bond in 98% (w/w) concentrated sulfuric acid based on the assessment of reactivity of four dipeptides at room temperature: l-alanyl-l-alanine (**1**), glycylglycine (**2**), glycyl-l-alanine (**3**), and l-alanylglycine (**4**). We find that dipeptides (**2**) and (**4**) are stable for at least two months in 98% w/w sulfuric acid, with no signs of reactivity. The dipeptides (**1**) and (**3**) are unstable and immediately begin complex solvolysis, which is mechanistically different from acid-catalyzed peptide bond hydrolysis. We show that the solvolysis of dipeptides (**1**) and (**3**) in 98% w/w sulfuric acid leads to the formation of alaninamide (**6**) and glicinamide (**7**), respectively. We propose that the mechanism of solvolysis of dipeptides (**1**) and (**3**) proceeds via dehydrogenation of the side chain methyl group (−CH_3_) of the C-terminal alanine. Consequently, the substitution of the −CH_3_ group of the C-terminal alanine with −CF_3_ stabilizes the l-alanyl-DL-trifluoroalanine dipeptide (**5**) to solvolysis in 98% w/w sulfuric acid.

## Introduction

The first experiments reporting sulfuric acid hydrolysis of proteins occurred more than 200 years ago when Braconnot isolated what we now know as glycine from animal tissue.^1^ For the following 130 years, studies continued to explore the reactivity of biological matter in concentrated sulfuric acid (and other acids). This early work sought to describe the chemical composition of biological material and eventually attempted to decipher the amino acid sequence in protein polymers. Such studies on the reactivity of proteins in concentrated sulfuric acid aimed to chemically cleave peptide bonds at specific amino acid positions in polypeptides (e.g., refs (2−6)). Despite decades of research, these early attempts at protein sequence analysis were generally unsuccessful. Today, we recognize that their procedure—treating the polypeptide chain with concentrated sulfuric acid at 4 °C or room temperature for several days (e.g., ref (7))—yields many unknown solvolysis products in concentrated sulfuric acid solution. These products would have been difficult to identify with the chemical analysis tools available at the time. The early approaches of chemical protein sequence analysis were eventually abandoned after the 1950 discovery and subsequent adoption of the Edman protein sequencing method.^8−10^

More than 200 years after the initial experiments on the sulfuric acid reactivity of proteins and peptides, we have revisited the mechanism of solvolysis of the peptide bond in 98% w/w aqueous concentrated sulfuric acid. We are motivated to revisit the reactivity of common biochemicals in this aggressive solvent, partially due to a renewed interest in understanding sulfuric acid chemistry in the context of the potential habitability of Venus's sulfuric acid clouds.^11−17^ The concentration of sulfuric acid in the clouds of Venus varies with altitude, from 81% w/w acid in the top clouds to 98% w/w acid in the lower cloud region.^18^ This concentration range is similar to the concentration of acid used in the early sulfuric acid reactivity studies mentioned above. We show by the identified reactivity products, alaninamide (**6**) and glycinamide (**7**), that the mechanism of peptide bond cleavage in 98% w/w sulfuric acid differs from the conventional acid-catalyzed hydrolysis mechanism known to occur in more diluted sulfuric acid (here at 81% w/w).^19,20^

## Results and Discussion

**Scheme 1:**
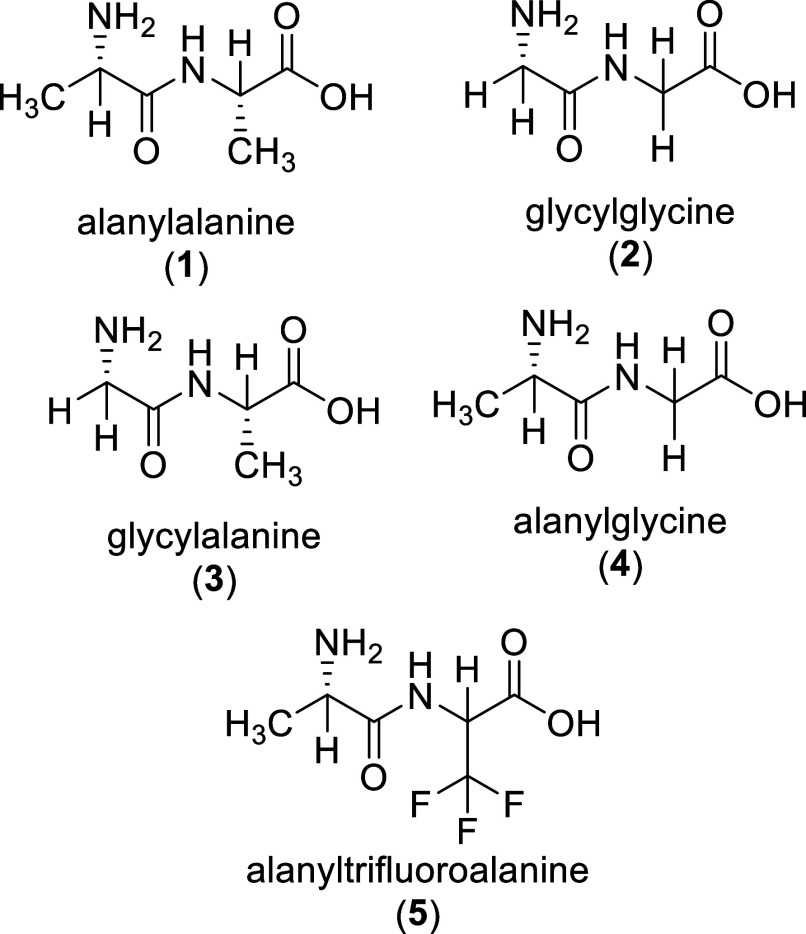
Dipeptides Tested in this Study All five compounds have been incubated in 81 and 98% w/w sulfuric acid or D_2_SO_4_ for NMR analysis, at room temperature (∼20 °C) for at least 1–2 months.

To investigate the stability and reactivity of the peptide bond in concentrated sulfuric acid, we studied four different dipeptides: l-alanyl-l-alanine (l-Ala-l-Ala, AA) (**1**), glycylglycine (Gly-Gly, GG) (**2**), glycyl-l-alanine (Gly-l-Ala, GA) (**3**), and l-alanylglycine (l-Ala-Gly, AG) (**4**) (Scheme 1). We chose dipeptides (**1**–**4**) as the simplest homodimer and nonhomodimer examples of peptides. Their structures are well-known and simple enough that their reactivity can be followed by NMR (Tables S1–S5), whereas interpreting data from more complex dipeptides or longer peptides would be much more challenging. We tested the reactivity of each of the dipeptides in aqueous concentrated sulfuric acid at two different concentrations (81% w/w and 98% w/w) at room temperature (∼20 °C). The conventional acid-catalyzed hydrolysis of the peptide bond in dilute acids depends on the presence of abundant water. The hydrolysis is accelerated by protonation of the carbonyl oxygen, followed by attack by water on the carbonyl carbon and the formation of the tetrahedral intermediate. The subsequent protonation of the amide nitrogen facilitates the cleavage of the peptide bond and the creation of carboxylic acid and amine groups of individual amino acids.^19,20^ In contrary to this established behavior of peptides in diluted acids, we found that the peptide bond stability in 98% w/w concentrated sulfuric acid, for peptides (**1**–**4**), falls into two categories, depending on the identity of the C-terminal amino acid.

**Figure 1:**
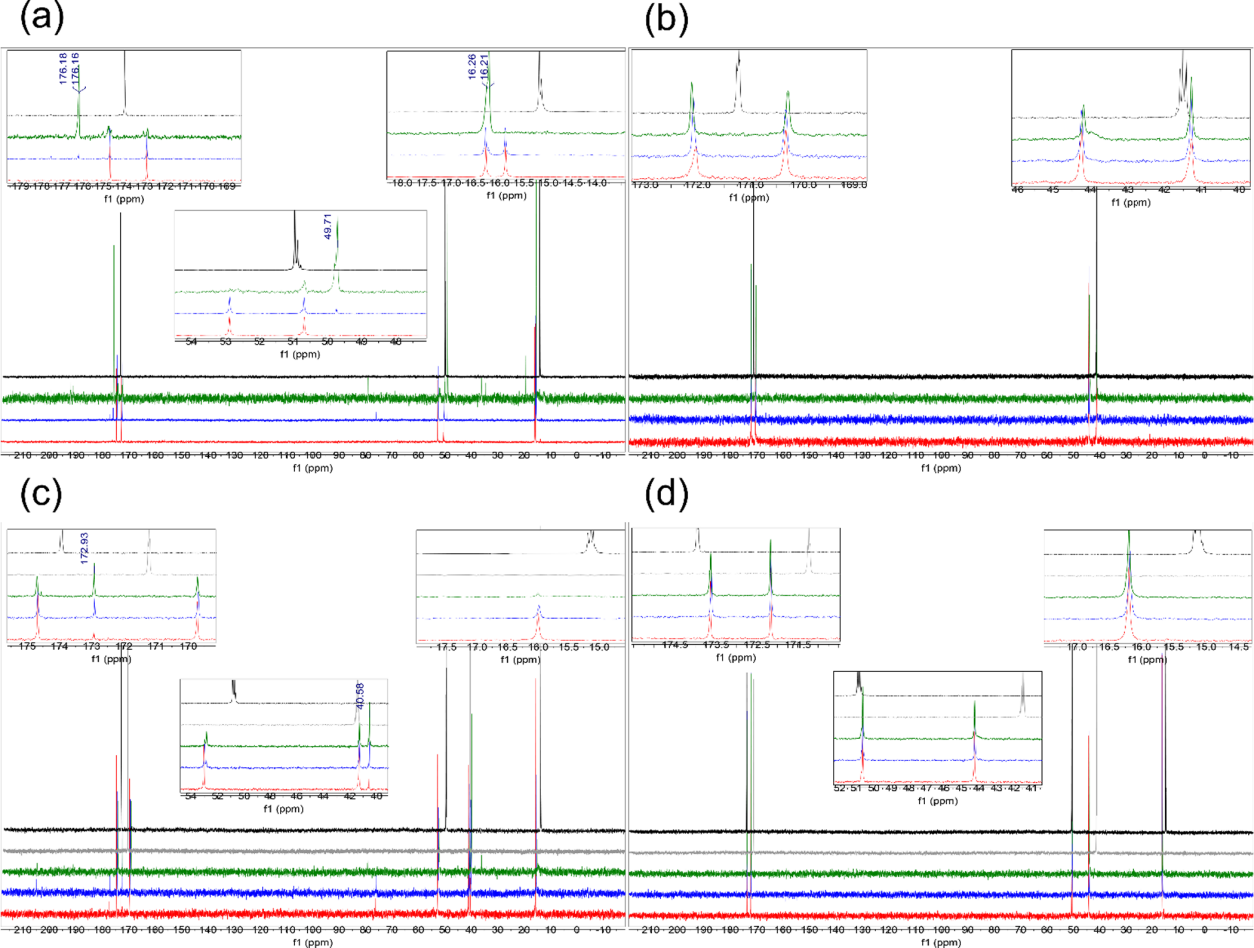
Comparison of the ^13^C NMR spectra for AA (**1**), GG (**2**), GA (**3**), and AG (**4**) in concentrated sulfuric acid (98% D_2_SO_4_ and 2% D_2_O, by weight), at room temperature. (a) Comparison of the ^13^C NMR of AA (**1**) collected after 1 day incubation (red spectra), 7 day incubation (blue spectra), and 2 month incubation (green spectra) to single amino acid alanine (black spectra).^12,21^ (b) Comparison of the ^13^C NMR of GG (**2**) collected after 1 day incubation (red spectra), 7 day incubation (blue spectra), and 2 month incubation (green spectra) to single amino acid glycine (black spectra).^12,21^ (c) Comparison of the ^13^C NMR of GA (**3**) collected after 1 day incubation (red spectra), 7 day incubation (blue spectra), and 1 month incubation (green spectra) to single amino acid, glycine (gray spectra) or alanine (black spectra). (d) Comparison of the ^13^C NMR of AG (**4**) collected after 1 day incubation (red spectra), 7 day incubation (blue spectra), and 1 month incubation (green spectra) to single amino acid, glycine (gray spectra) or alanine (black spectra). The GG (**2**) and AG (**4**) are stable in 98% w/w D_2_SO_4_, while AA (**1**) and GA (**3**) undergo solvolysis, leading to different products (ppm values shown) than the acid-catalyzed hydrolysis.

**Figure 2:**
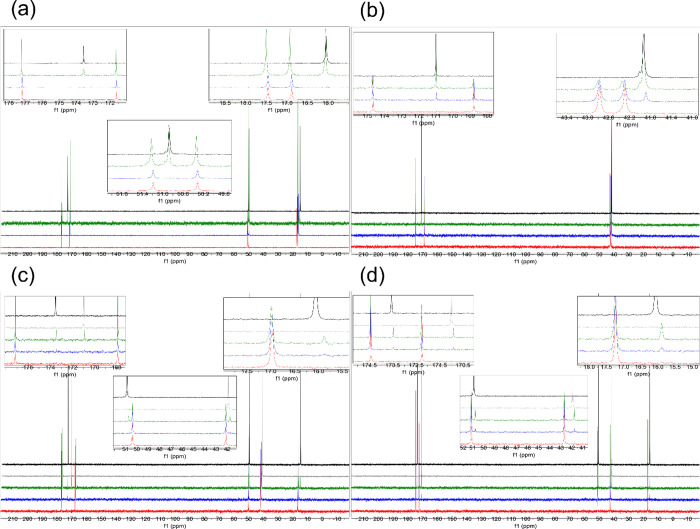
Comparison of the ^13^C NMR spectra for AA (**1**), GG (**2**), GA (**3**), and AG (**4**) in concentrated sulfuric acid (81% D_2_SO_4_ and 19% D_2_O, by weight), at room temperature. (a) Comparison of the ^13^C NMR of AA (**1**) collected after 1 day incubation (red spectra), 7 day incubation (blue spectra), and 2 month incubation (green spectra) to single amino acid alanine (black spectra).^12,21^ (b) Comparison of the ^13^C NMR of GG (**2**) collected after 1 day incubation (red spectra), 7 day incubation (blue spectra), and 2 month incubation (green spectra) to single amino acid glycine (black spectra).^12,21^ (c) Comparison of the ^13^C NMR of GA (**3**) collected after 1 day incubation (red spectra), 7 day incubation (blue spectra), and 1 month incubation (green spectra) to single amino acid, glycine (gray spectra) or alanine (black spectra). (d) Comparison of the ^13^C NMR of AG (**4**) collected after 1 day incubation (red spectra), 7 day incubation (blue spectra), and 1 month incubation (green spectra) to single amino acid, glycine (gray spectra) or alanine (black spectra). All four tested dipeptides undergo the conventional acid-catalyzed hydrolysis of the peptide bond with the release of monomeric amino acids.

The GG and AG dipeptides appear to be completely stable for at least 2 months in 98% (w/w) sulfuric acid at room temperature, with no signs of reactivity (Figures 1, S1, S4, S6, S8, S9). This differs from the conventional peptide bond acid-catalyzed hydrolysis in more dilute acidic solutions (Figures 2, S2 and S7). In contrast, the AA and GA dipeptides are unstable in 98% w/w sulfuric acid and undergo complex reactivity that does not result in the release of the individual amino acids, as occurs in the conventional hydrolysis of the peptide bond. The reactivity of AA and GA in 98% w/w sulfuric acid does result in the breakage of the dipeptide, with products distinct from the individual amino acids that form the reactive dipeptides (Figure 1). The 2D ^1^H–^15^N HMBC NMR experiments support this result as well (Figures S3–S6). The 2D NMR also rules out a dehydration cyclization product as a major product in 98% w/w sulfuric acid (see SI). Finally, to further demonstrate that the dominant solvolysis products in 98% w/w sulfuric acid are not unmodified monomeric amino acids, we have incubated the AA dipeptide for 7 days in 98% w/w sulfuric acid, followed by spiking the sample with individual l-Ala amino acid. The ^13^C and ^1^H NMR spectra of the spiked sample show that the peaks corresponding to the spiked l-Ala amino acid do not overlap with the peaks of the dominant solvolysis product. This result further confirms that single, unmodified alanine is not the product of the solvolysis of the AA dipeptide in 98% (w/w) sulfuric acid (Figures S10 and S11). The same experiment, repeated in 81% w/w sulfuric acid, shows overlapping peaks of the spiked l-Ala amino acid with the peaks of the hydrolysis product, further confirming that in 81% w/w acid, the AA dipeptide hydrolyzes to individual single alanine residues (Figures S12 and S13).

**Figure 3:**
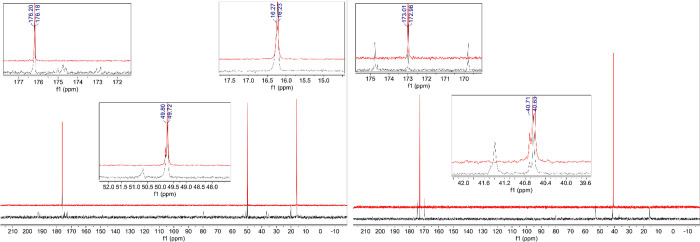
Comparison of the ^13^C NMR of AA (**1**) and GA (**3**) collected after 2 month incubation in concentrated sulfuric acid (98% D_2_SO_4_ and 2% D_2_O, by weight), at room temperature (black spectra), to the spectra of alaninamide (**6**) (red spectra) (*left panel*) and glycinamide (**7**) (red spectra) (*right panel*). The comparison shows that the alaninamide and glycinamide spectra overlap with the spectra of the dominant solvolysis products of the AA and GA dipeptides, respectively. The results confirm that alaninamide and glycinamide are the products of the solvolysis of the AA and GA dipeptides, respectively, in 98% (w/w) sulfuric acid.

We now turn to the identification of the chemicals that could be the dominant products of the solvolysis of the AA and GA dipeptides. One of the possible products of the solvolysis of the AA and GA dipeptides in 98% w/w sulfuric acid is the amide variants of amino acids alanine and glycine—alaninamide (**6**) and glycinamide (**7**), respectively (Table S6). To demonstrate that the dominant solvolysis products of AA and GA in 98% w/w sulfuric acid are amide-modified amino acids, we compared the ^1^H and ^13^C NMR spectra of alaninamide and glycinamide collected in 98% w/w sulfuric acid to the spectra of AA and GA dipeptides incubated for two months in 98% w/w sulfuric acid at room temperature. The spectral comparison shows that the alaninamide and glycinamide NMR peaks overlap with the spectra of the solvolysis products of the AA and GA dipeptides (Figure 3). These results confirm that alaninamide (**6**) and glycinamide (**7**) are the most likely products of the solvolysis of the AA and GA dipeptides in 98% (w/w) sulfuric acid. To further demonstrate that the dominant solvolysis products in 98% w/w sulfuric acid are indeed alaninamide (**6**) and glycinamide (**7**), we incubated the AA and GA dipeptides in 98% w/w sulfuric acid, followed by spiking the sample with alaninamide (**6**) and glycinamide (**7**), respectively. The ^13^C and ^1^H NMR spectra of the spiked sample show that the peaks corresponding to the spiked amide variants of amino acids do overlap with the peaks of the dominant solvolysis products of AA and GA. This result further confirms that alaninamide (**6**) and glycinamide (**7**) are the dominant products of the solvolysis of the AA and GA dipeptides in 98% w/w sulfuric acid (Figures S15 and S16).

**Scheme 2:**
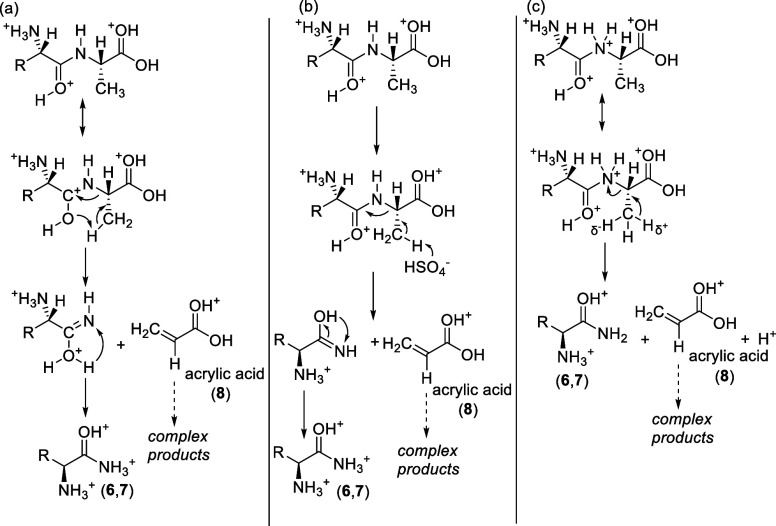
Proposed Solvolysis Reaction Mechanism for AA and GA Dipeptides in 98% w/w Concentrated Sulfuric Acid In all three variants of the reaction, the solvolysis proceeds via the dehydrogenation of the side chain methyl group (−CH_3_) of the C-terminal alanine. The dominant products of the reaction are alaninamide (**6**) and glycinamide (**7**) for AA and GA dipeptides, respectively, and acrylic acid (**8**) that gives rise to the reactive byproducts. R = H, CH_3_ in GA and AA, respectively.

We now propose three possible variants of the mechanism of the peptide bond solvolysis in 98% w/w sulfuric acid (Scheme 2). In all three cases, the carbonyl oxygens of the dipeptide are fully protonated in concentrated sulfuric acid (the complete protonation of the peptide carbonyl oxygens in concentrated sulfuric acid has been a well-known fact for decades^22−24^). In each case, the solvolysis proceeds via the dehydrogenation of the side chain methyl group (−CH_3_) of the C-terminal alanine. The difference between the three proposed reaction mechanisms depends on the nature of the chemical moiety that polarizes the C–H bond of the −CH_3_ group and primes it for the dehydrogenation reaction.

The first mechanism (Scheme 2a) relies on the tautomeric rearrangement in the dipeptide and the protonation of the oxygen of the peptide carbonyl group in 98% (w/w) sulfuric acid. The protonation of the carbonyl polarizes the C–H bond in −CH_3_ of the C-terminal alanine and primes the side chain methyl group for the dehydrogenation reaction and the subsequent breakage of the C–N bond in the C-terminal alanine. We note one potential weakness of the first mechanism. In concentrated H_2_SO_4_, we might expect the intermediate −C–OH_2_^+^ group to dehydrate to the reactive C^+^ group and H_2_O. Whether such a dehydration reaction can really happen is unknown.

The second mechanism (Scheme 2b) relies on the direct attack of the sulfuric acid HSO_4_^–^ ion on the C–H group of the C-terminal alanine methyl side chain. However, several observations speak against this possibility. First, the concentration of the HSO_4_^–^ ion in 98% w/w sulfuric acid is very low and certainly much lower than in 81% w/w acid.^25^ The low abundance of the HSO_4_^–^ ion could limit its ability to polarize the C–H bond of the methyl group. Second, if the HSO_4_^–^ ion primes the dehydrogenation reaction in the AA and GA dipeptides, we would expect similar reactions happening in single amino acids that have alkyl side chain groups. We do not see any evidence of such reactivity. All individual alkyl side chain amino acids appear to be stable in 98% w/w sulfuric acid for many weeks, if not longer, with no sign of reactivity.^12^

The third mechanism variant (Scheme 2c) relies on the protonation of the amide nitrogen of the peptide bond, in addition to the expected protonation of the carbonyl oxygen. The protonation of the amide nitrogen could only happen in a very strong acid. In this scenario, the protonated carbonyl oxygen of the peptide bond pulls electrons from the methyl group and hence induces a dipole that predisposes the C–H proton to break away from the −CH_3_ group, resulting in the dehydrogenation reaction. We do not know if the peptide amide group is protonated in 98% w/w acid; it is however in principle possible, and it would also further explain the difference between the reactivity of the dipeptides in 98 and 81% w/w sulfuric acid (where the amide nitrogen is likely not stably protonated).

The analogous dehydrogenation reaction cannot happen to the GG and AG dipeptides via any of the proposed mechanisms. Due to the lack of the alkyl side chain group, the dehydrogenation reaction cannot proceed if the C-terminal amino acid is glycine, i.e., in the GG and AG dipeptides, which explains their stability in 98% w/w sulfuric acid.

We propose that the N-terminal, amide derivative of the amino acid alanine, alaninamide (**6**), is the main stable product of the dehydrogenation solvolysis reaction of the AA dipeptide in 98% w/w concentrated sulfuric acid (Figure 3). Likewise, the reactive GA dipeptide follows an analogous mechanism, with the release of glycinamide (**7**) (Scheme 2) (Figure 3). Long-term incubation results suggest that the single chemical species products giving the dominant NMR signal are stable to further reactivity in 98% w/w concentrated sulfuric acid (Figure S9). The unstable C-terminal component of the dipeptide, acrylic acid (**8**), appears to undergo complex reactivity and changes over time, eventually resulting in the formation of a complex mixture of products analogous to “red oil”.^26−28^

As a control, we studied all four dipeptides (AA, GG, GA, and AG) in 81% w/w aqueous sulfuric acid at room temperature. All four tested dipeptides appear to undergo the conventional acid-catalyzed hydrolysis of the peptide bond that depends on the presence of abundant water (Figures 2, S2, S7, S12, S13).^19,20^ Consistent with hydrolysis, this reactivity results in the release of the monomeric amino acids that form the dipeptide. We note that the efficiency of the hydrolysis varies based on the dipeptide's amino acid composition (Figure 2). The released monomeric amino acids are stable in 81% w/w sulfuric acid and do not undergo further reactivity.^12^

The proposed solvolysis mechanism is supported by NMR experiments. As explained by our reaction mechanism, the GG and AG dipeptides are entirely stable to solvolysis in 98% w/w concentrated sulfuric acid. This result is supported by ^1^H and ^13^C NMR data, which have not changed from 1 day to 1 week to two months (Figures 1 and S1). The stability is also supported by 2D ^1^H–^15^N NMR, which shows evidence for the intact peptide bond on the above time scales (Figures S3–S6).

The mechanism also explains the observation that AA and GA are unstable to solvolysis in 98% (w/w) concentrated sulfuric acid. The first supporting point is the NMR, which shows that AA and GA have not broken down to their individual amino acid components (^1^H and ^13^C NMR in Figures 1 and S1). The second supporting point is that ^13^C NMR spectra are consistent with alaninamide (**6**) (three dominant carbon peaks that overlap well with the reference ^13^C NMR spectrum of alaninamide, Figure 3a) and glycinamide (**7**) (two dominant carbon peaks that overlap well with the reference ^13^C NMR spectrum of glycinamide, Figure 3b). The ^13^C NMR peaks of the dominant products of solvolysis are shifted from the alanine and glycine carbon peaks, and the shift magnitude is consistent with the amide modification of the amino acids. Finally, the solutions of AA and GA in 98% w/w concentrated sulfuric acid turn yellow immediately and dark red to brown after a couple of months, while GG and AG remain clear (Figure S8). The coloration of the solution comes from the reactive complex products that arise from the reactivity of the acrylic acid (**8**) in 98% w/w sulfuric acid. The dark red color is commonly known to result from byproducts of the reactions, colloquially called “red oil”, between various organic molecules in concentrated sulfuric acid.^26−28^

The proposed solvolysis reaction should happen exclusively in highly concentrated sulfuric acid, where there is no abundant water (e.g., 98% w/w) and only to dipeptides that have the C-terminal amino acids with alkyl side chain groups (i.e., −CH_2_– or −CH_3_) bonded to the C-α carbon (like in AA and GA). Only in such a structural context can alanine’s −CH_3_ group be primed for a dehydrogenation reaction. GG and AG dipeptides, with the C-terminal glycine, cannot undergo the analogous dehydrogenation reaction and are stable to solvolysis in 98% (w/w) sulfuric acid. We therefore expect this mechanism of solvolysis to apply to peptide bonds with any of the amino acids found in proteins, other than glycine in the C-terminal position. Initial results from the solvolysis of 20 homodipeptides support this conclusion.^21^

**Figure 4:**
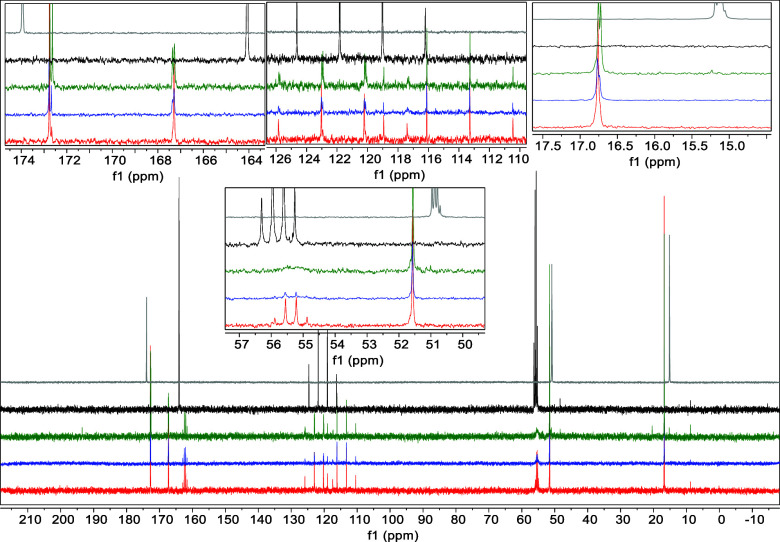
Comparison of the ^13^C NMR spectra for A3FA (**5**), in concentrated sulfuric acid (98% D_2_SO_4_ and 2% D_2_O, by weight) at room temperature. Comparison of the ^13^C NMR of A3FA (**5**) collected after 1 day incubation (red spectra), 7 day incubation (blue spectra), and 1 month incubation (green spectra) to single amino acid trifluoroalanine (black spectra) or alanine (gray spectra).^12,21^ The A3FA (**5**) is stable in 98% w/w D_2_SO_4_ for at least 1 month. It should be noted that the additional quadruplet peaks around 162 and 115 ppm come from the contaminant trifluoroacetic acid (TFA) that is used during the synthesis procedure.

**Figure 5:**
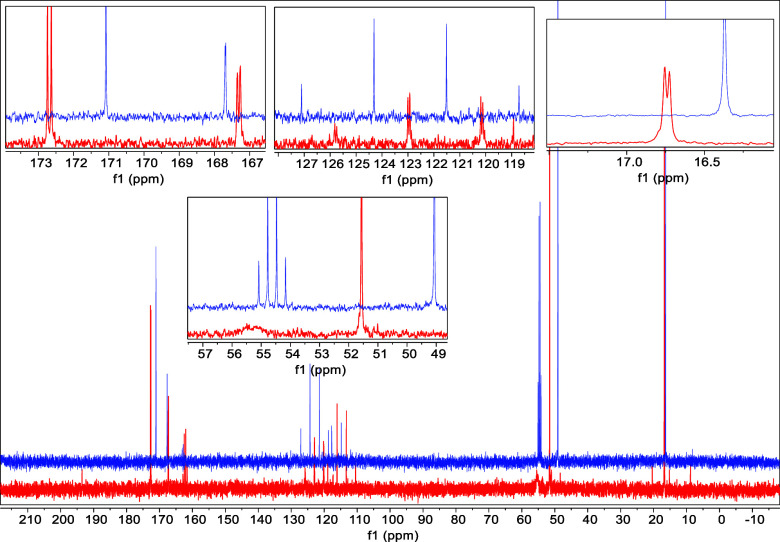
^13^C NMR spectra of the A3FA dipeptide (**5**) in 98% w/w (red spectra) collected after 1 month of incubation compared to the ^13^C NMR spectra collected in D_2_O (blue spectra). The spectral peak shifts for 98% w/w D_2_SO_4_ largely agree with the chemical shift values in D_2_O, further supporting the stability of the A3FA dipeptide in 98% w/w sulfuric acid. It should be noted that the additional quadruplet peaks around 162 and 115 ppm come from the contaminant trifluoroacetic acid (TFA) that is used during the synthesis procedure.

To confirm the key role of the side chain C–H group in the solvolysis reaction, we synthesized a fluorine-containing variant of the AA (**1**) dipeptide, l-alanyl-DL-trifluoroalanine (A3FA; (**5**)). The −CF_3_ group of A3FA cannot undergo the dehydrogenation reaction, which should stabilize the dipeptide in 98% w/w sulfuric acid. Indeed, in contrast to the AA solution (Figure S8), the A3FA solution remains clear during the 1 month incubation in 98% w/w sulfuric acid, with no signs of reactivity (Figure S21). The ^13^C NMR data confirm our prediction and support the proposed dehydrogenation solvolysis mechanism (Scheme 2). The substitution of the −CH_3_ group of the C-terminal alanine with −CF_3_ stabilizes the dipeptide (**5**) to solvolysis in 98% w/w sulfuric acid for at least 1 month (Figures 4 and 5; see also Figures S17–S21 in the SI). We note that over time, the ^13^C NMR signal corresponding to the C2 alpha-carbon in the A3FA dipeptide splits and broadens (Figure 4). The splitting and broadening of the C2 peak could indicate efficient exchange of the C2 proton of the dipeptide with the solvent’s deuterium (i.e., H/D exchange) and is not a sign of instability of the compound.

In summary, our results confirm that alaninamide and glycinamide are the dominant products of solvolysis of AA and GA dipeptides in 98% (w/w) sulfuric acid and establish that the dehydrogenation of the alanine side chain is the key step in the solvolysis reaction mechanism.

## Supporting Information Available

Assignments of carbon and hydrogen atoms of the AA, GG, GA, AG and A3FA dipeptides, additional support for the presented solvolysis mechanism in 98% w/w sulfuric acid, and methods (PDF)
