# Supplementary material for: Mechanism for Peptide Bond Solvolysis in 98% w/w Concentrated Sulfuric Acid
Source: ACS Omega. 2025 Jan 22;10(9):9623–9. doi: 10.1021/acsomega.4c10873 (PMC11904706; doi:10.1021/acsomega.4c10873)
Supplement: Supplementary file 1 — ao4c10873_si_001.pdf [file ao4c10873_si_001.pdf]

Supplementary Information

A Mechanism for Peptide Bond Solvolysis in 98% w/w Concentrated Sulfuric Acid

Janusz J. Petkowski[1,2‡,*], Maxwell D. Seager[3,4,‡], William Bains[5,6], John H. Grimes Jr.[7], Sara Seager[4,8,9,10‡]

[1] Faculty of Environmental Engineering, Wroclaw University of Science and Technology, 50-370 Wroclaw, Poland

[2] JJ Scientific, Mazowieckie, Warsaw 02-792, Poland.

[3] Department of Chemistry and Biochemistry, Worcester Polytechnic Institute, Worcester, MA 01609, USA

[4] Nanoplanet Consulting, Concord, MA 01742, USA

[5] School of Physics & Astronomy, Cardiff University, 4 The Parade, Cardiff CF24 3AA, UK

[6] Rufus Scientific, Melbourn, Herts SG8 6ED, UK

[7] Complex Carbohydrate Research Center, University of Georgia, 315 Riverbend Road, Athens, GA 30602, USA

[8] Department of Earth, Atmospheric and Planetary Sciences, Massachusetts Institute of Technology, 77 Massachusetts Avenue, Cambridge, MA 02139, USA

[9] Department of Physics, Massachusetts Institute of Technology, 77 Massachusetts Avenue, Cambridge, MA 02139, USA

[10] Department of Aeronautics and Astronautics, Massachusetts Institute of Technology, 77 Massachusetts Avenue, Cambridge, MA 02139, USA

‡ These authors contributed equally.

* Correspondence: Janusz J. Petkowski: janusz.petkowski@pwr.edu.pl

Supplementary Information Text

In this Supplementary Information file, we present additional experimental data that go beyond the ^{13}C NMR presented in the main text. The goal is to provide detailed experimental results to further support the proposed mechanism of solvolysis of the peptide bond in 98% w/w sulfuric acid described in the main paper (see Section S2 below). We present results from the ^{13}C NMR and ^{1}H NMR experiments (Figure S1-S2) and 2D ^{1}H-^{15}N Heteronuclear Multiple Bond Correlation spectroscopy (HMBC) NMR (Figure S3-S6) experiments to further demonstrate the stability and reactivity of the peptide bond in the sulfuric acid concentrations (81% w/w and 98% w/w, the rest water) for the four tested dipeptides (**1**-**4**).

We assign the carbon and hydrogen peaks by comparison with literature data collected in D_2O[1] (Figure S7) and by our 2D ^{1}H-^{15}N HMBC NMR experiments (Figures S3-S6) (see Section S1).

S1. The assignments of carbon and hydrogen atoms of the AA, GG, GA, AG and A3FA dipeptides.

We assign the carbon and hydrogen peaks of the four tested dipeptides (**1**-**4**) by comparison of the data collected after 1 day incubation in 98% w/w with literature data collected in D_2O[1] (Figure S7; Table S1-S4). We further confirm the assignments of the dipeptide hydrogen atoms by our 2D ^{1}H-^{15}N HMBC NMR experiments (Figures S3-S6). We also assign the carbon and hydrogen peaks of the A3FA (**5**) dipeptide in 98% w/w D_2SO_4 and D_2O (Table S5). We note that, as it was in the case of single amino acids [2] and nucleic acid bases [3,4], the nitrogen atoms of free amino groups as well as carbonyl oxygens are likely fully protonated in concentrated sulfuric acid [5,6].

S2. Additional support for the presented solvolysis mechanism in 98% w/w sulfuric acid.

We use the results of our NMR experiments, supported by the literature data, as a guide in the elucidation of the possible mechanism of solvolysis of the peptide bond in 98% w/w sulfuric acid.

Below we discuss the results of the AA (**1**) NMR experiments in detail, emphasizing the support of the data and the available literature for the proposed mechanism. We note that the other reactive dipeptide, GA (**3**) follows the same solvolysis mechanism.

We first discuss the possible dominant products that emerge after 2-month incubation of the AA and GA dipeptides in 98% w/w sulfuric acid (Table S6).

After 2-month incubation of the AA dipeptide in 98% w/w sulfuric acid three dominant peaks emerge on the ^{13}C NMR spectrum (Figure 1). The three dominant peaks suggest that the dominant solvolysis product is a three-carbon molecule, with a carbonyl group (peak at 176.18 ppm, that is more deshielded than the original carboxylic group of the AA dipeptide), an α-carbon (peak at 49.71 ppm) and a methyl group carbon (peak at 16.21 ppm). The emergent peaks are close to, but do not overlap with, the single alanine spectrum collected in 98% w/w sulfuric acid (Figure S10). The lack of overlap suggests that the dominant product that emerges after 2 month incubation in acid is not the original amino acid but rather a molecule that is structurally similar to alanine.

The modified alanine, as a dominant product, likely originates from the N-terminal amino acid of the dipeptide. The diverse minor products arising from the reactivity of the C-terminal alanine are the source of the small additional peaks visible on the ^{13}C NMR spectra, and are responsible for the dark reddish-brown color of the AA and GA reaction mixtures (Figure S8). This conclusion is supported by the observation that the dominant product of the GA dipeptide, that appears to undergo analogous solvolysis reaction to AA has two dominant peaks on the ^{13}C NMR spectrum, suggesting that in the case of solvolysis of GA the dominant product is a modified glycine residue (Figure 1).

We have identified the most likely candidate for the dominant product of the solvolysis of AA and GA dipeptides in 98% w/w sulfuric acid – alaninamide (**6**) and glycinamide (**7**) respectively, based on comparison of the ^{1}H and ^{13}C NMR spectra (Figure 3, Figures S15-S16). We have also ruled out several other potential molecules as candidates for the solvolysis products (Table S6). We describe our rationale for ruling them out briefly below.

The lack of the nitrogen signal for the amide group on the 2D ^{1}H-^{15}N HMBC NMR spectra (Figure S3) rules out a dehydration cyclization product as a major product of solvolysis in 98% w/w sulfuric acid. Such cyclic AA product (3,6-dimethylpiperazine-2,5-dione) would also result in three dominant peaks on the ^{13}C NMR spectrum, due to symmetric nature of the cyclic molecule, but it would be expected to give a distinct ^{1}H-^{15}N HMBC signal in the amide region of the NMR spectrum [7], which we do not observe (Figure S3). Similarly, we do not see such cyclization for GG and AG dipeptides in 98% w/w sulfuric acid. If it happens, we would expect the dehydration and cyclization reactivity to affect all tested dipeptides in a similar way. Meanwhile GG and AG dipeptides are completely stable and unchanged (Figure 1), with clearly visible 2D ^{1}H-^{15}N HMBC NMR signals of intact peptide bonds after 2 month incubation in 98% w/w sulfuric acid (Figure S4 and Figure S6).

We have also ruled out the N-terminally sulfated alanine as a candidate for the dominant product of the solvolysis. The N-terminal amino group could become sulfated in 98% w/w concentrated sulfuric acid. The N-sulfation of amino acids (i.e. the formation of sulfamates) has been postulated by some of the early studies on the reactivity of proteins and peptides in concentrated sulfuric acid [8,9]. Such modification however has never been confirmed and other early studies do not support it [10]. Our ^{1}H-^{15}N HMBC NMR experiments also do not support the N-sulfation of the α-amino group. The HMBC correlation for the free amino group does not undergo significant change in chemical shift between 1 day and 2 months, and a significant chemical shift change is expected if the α-amino group was N-sulfated (Figure S3 and Figure S6).

Another possibility is the stable sulfation of the carboxylic group of the N-terminal amino acid. The shift of the carbonyl peak of the dominant product towards higher ppm (from 172.87 ppm to 176.18 ppm) could be in principle consistent with the sulfation of the carbonyl group. The sulfation of the carboxylic oxygen and the formation of carboxylic sulfuric anhydrides occurs in concentrated sulfuric acid (e.g. [11]) although not all carboxylic acids are sulfated in concentrated sulfuric acid and when it happens, sulfation does not affect all carboxylic acids uniformly [12]. Upon sulfation some carboxylic acids rearrange into other sulfated species (e.g. acetic sulfuric anhydride) [11,13,14], while other compounds are more stable [15–17]. We note however that we see no evidence of sulfation of carboxylic oxygen in amino acids [2].

S3. Supplementary Materials and Methods.

We purchased a set of four L-amino acid dipeptides from Millipore-Sigma with $\geq$ 98% purity (catalog numbers: Glycylglycine: G1002, Glycyl-L-alanine: 50150, L-Alanyl-L-alanine: A9502, L-Alanyl-glycine: A0878). The alaninamide hydrochloride, with $\geq$ 95% purity, comes from Fisher Scientific Co. LCC (catalog number: AAH6301906). The glycinamide hydrochloride, with $\geq$ 98% purity, comes from Millipore-Sigma (catalog number: 50070). The trifluoroacetate (TFA) salt of the L-alanyl-DL-trifluoroalanine dipeptide was custom synthesized, with $\geq$ 95% purity, by Pierce Custom Peptides Thermofisher Life Technologies Corporation. We used D_2SO_4 from ACROS Organics (sulfuric acid-d_2 for NMR, 98 wt.% in D_2O, 99.5+ atom % D) and D_2O (deuteration degree min 99.9%) from MagniSolv.

We prepared our NMR samples by dissolving 25 to 85 mg of the dipeptides into 500 μL of solvent D_2SO_4 in D_2O in glass vials. We used 10 to 40 mg of compounds for the 1D ^1H and ^{13}C NMR. We used 25 to 85 mg for some of the 2D NMR. When required, we have heated sealed glass vials in a hot water bath (~80 ºC for a few minutes) to promote dissolution of the compounds. We transferred the solution to 5 mm NMR tubes and stored the tubes for 12 to 18 hours before NMR measurements. After NMR measurements we stored the solutions in the NMR tubes, where the storage room temperature varied from about 18 to 24 ºC.

For the “spiking” 1D ^1H and ^{13}C NMR experiment we used 30 mg of the AA (**1**) dipeptide dissolved in 700 μL of solvent D_2SO_4 in D_2O (either 98% w/w or 81% w/w D_2SO_4 in D_2O). We incubated the AA dipeptide for 7 days in 98% w/w or 81% w/w sulfuric acid followed by spiking the sample with 30 mg of single L-Ala amino acid and subsequent NMR re-measurement of the same sample.

For the “spiking” 1D ^1H and ^{13}C NMR experiment we used 30 mg of the AA (**1**) and GA (**3**) dipeptide dissolved in 700 μL of solvent D_2SO_4 in D_2O (98% w/w D_2SO_4 in D_2O). We incubated the AA and GA dipeptides for 7 days in 98% w/w sulfuric acid followed by spiking the sample with 30 mg of alaninamide and glycinamide respectively and subsequent incubation of the sample for another 10 weeks before the re-measurement of the same sample.

To acquire NMR data, we used a Bruker Avance III-HD 400 MHz spectrometer equipped with a Prodigy liquid nitrogen cryoprobe (BBO) at 25 °C. We acquired 1D ^1H, ^{13}C, and 2D ^1H-^{15}N HMBC NMR spectra to confirm the structures and hence stability of the compounds in 98% w/w and 81% w/w D_2SO_4 in D_2O. In all cases we locked on D_2SO_4. The D_2SO_4 peak is at 11.48 +/- 0.02 ppm in 98% w/w D_2SO_4 and at 11.99 +/- 0.02 ppm in 81% w/w D_2SO_4.

We used MNova software (Mestrelab Research) to process and analyze the NMR data[18]. The original data for all NMR experiments are available for download as Supplementary Datasets from Zenodo at https://zenodo.org/records/11122291.

Supplementary Datasets:

The NMR measurements in sulfuric acid at different concentrations follows the following naming convention: different concentrations (by weight) of sulfuric acid in water are denoted by two different letters "H" and "K": K refers to 98% D_2SO_4/2% D_2O and H refers to 81% D_2SO_4/19% D_2O at room temperature. The original NMR file names that contain the letter "A" denote the NMR spectra that are collected in pure D_2O.

Supplementary Dataset S1: The original 1D ^{1}H and ^{13}C NMR data collected in 81% w/w and 98% w/w D_2SO_4 in D_2O, at all three time intervals, for all four AA (**1**), GG (**2**), GA (**3**) and AG (**4**) dipeptides. The reference data for single, monomeric amino acids, L-alanine and glycine come from the work of Seager et al. [2]. All data can be downloaded from Zenodo at https://zenodo.org/records/11122291.

Supplementary Dataset S2: The original 2D ^{1}H-^{15}N HMBC NMR data for all four AA (**1**), GG (**2**), GA (**3**) and AG (**4**) dipeptides collected in 98% w/w D_2SO_4 in D_2O. All data can be downloaded from Zenodo at https://zenodo.org/records/11122291.

Supplementary Dataset S3: The original 1D ^{1}H and ^{13}C NMR data for the AA (**1**) dipeptide spiked with single individual L-Ala amino acid collected in 81% w/w and 98% w/w D_2SO_4 in D_2O. All data can be downloaded from Zenodo at https://zenodo.org/records/11122291.

Supplementary Dataset S4: The original 1D ^{1}H and ^{13}C NMR data collected in 98% w/w D_2SO_4 in D_2O for alaninamide and glycinamide. The dataset also includes the original 1D ^{1}H and ^{13}C NMR data for the AA (**1**) and GA (**3**) dipeptides spiked with alaninamide and glycinamide collected in 98% w/w D_2SO_4 in D_2O. All data can be downloaded from Zenodo at https://zenodo.org/records/11122291.

Supplementary Dataset S5: The original 1D ^{1}H, ^{13}C NMR and ^{19}F NMR data for the A3FA (**5**) dipeptide and the individual trifluoroalanine amino acid collected in 81% w/w and 98% w/w D_2SO_4 in D_2O, as well as D_2O. All data can be downloaded from Zenodo at https://zenodo.org/records/11122291.

Supplementary Figures.

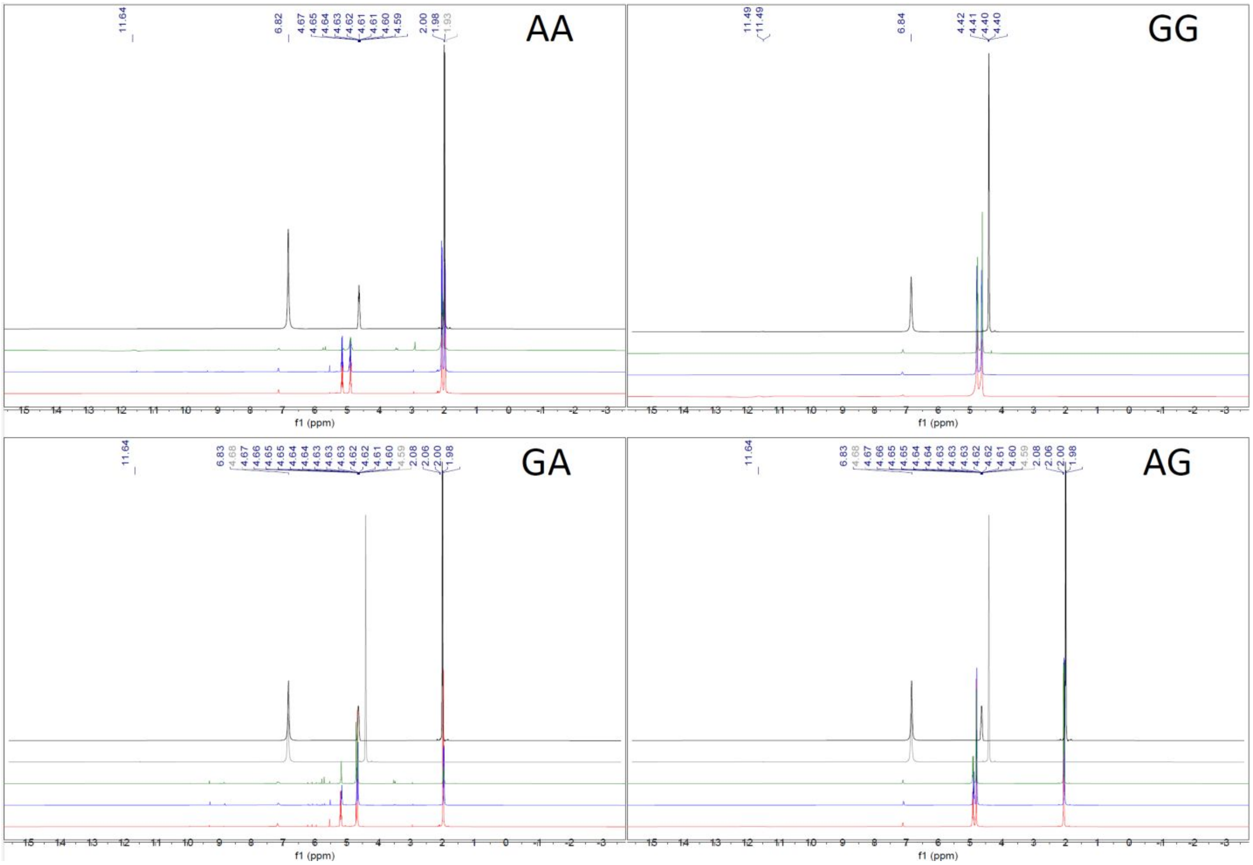

Figure S1. The comparison of the ^{1}H NMR spectra for AA, GG, GA, AG in concentrated sulfuric acid (98% D_2SO_4 and 2% D_2O, by weight), at room temperature, collected after 1 day incubation, 7 day incubation to spectra collected after 1-2 month incubation and compared to single amino acid, glycine or alanine. The solvent signal is suppressed for clarity. The spectra are color coded as in Figure 1 and 2 in the main text. **A)** The ^{1}H NMR of AA. **B)** The ^{1}H NMR of GG. **C)** The ^{1}H NMR of GA. **D)** The ^{1}H NMR of AG. The GG (**2**) and AG (**4**) are stable in 98% w/w D_2SO_4 while AA (**1**) and GA (**3**) undergo solvolysis without the release of the native monomeric amino acids.

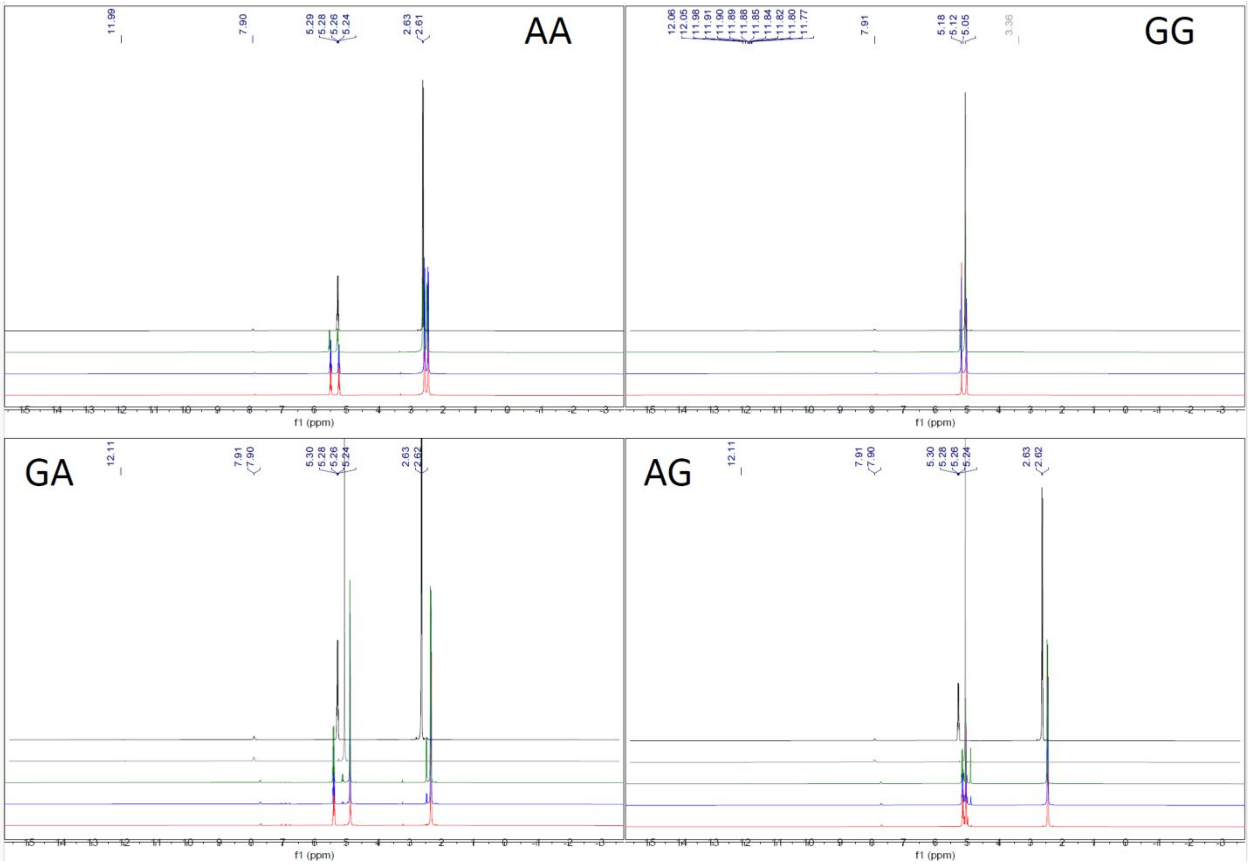

Figure S2. The comparison of the ^1H NMR spectra for AA, GG, GA, AG in concentrated sulfuric acid (81% D_2SO_4 and 19% D_2O, by weight), at room temperature, collected after 1 day incubation, 7 day incubation to spectra collected after 1-2 month incubation and compared to single amino acid, glycine or alanine. The spectra are color coded as in Figure 1 and 2 in the main text. The solvent signal is suppressed for clarity. **A)** The ^1H NMR of AA. **B)** The ^1H NMR of GG. **C)** The ^1H NMR of GA. **D)** The ^1H NMR of AG. All four tested dipeptides undergo the conventional acid-catalyzed hydrolysis of the peptide bond with the release of the single amino acids.

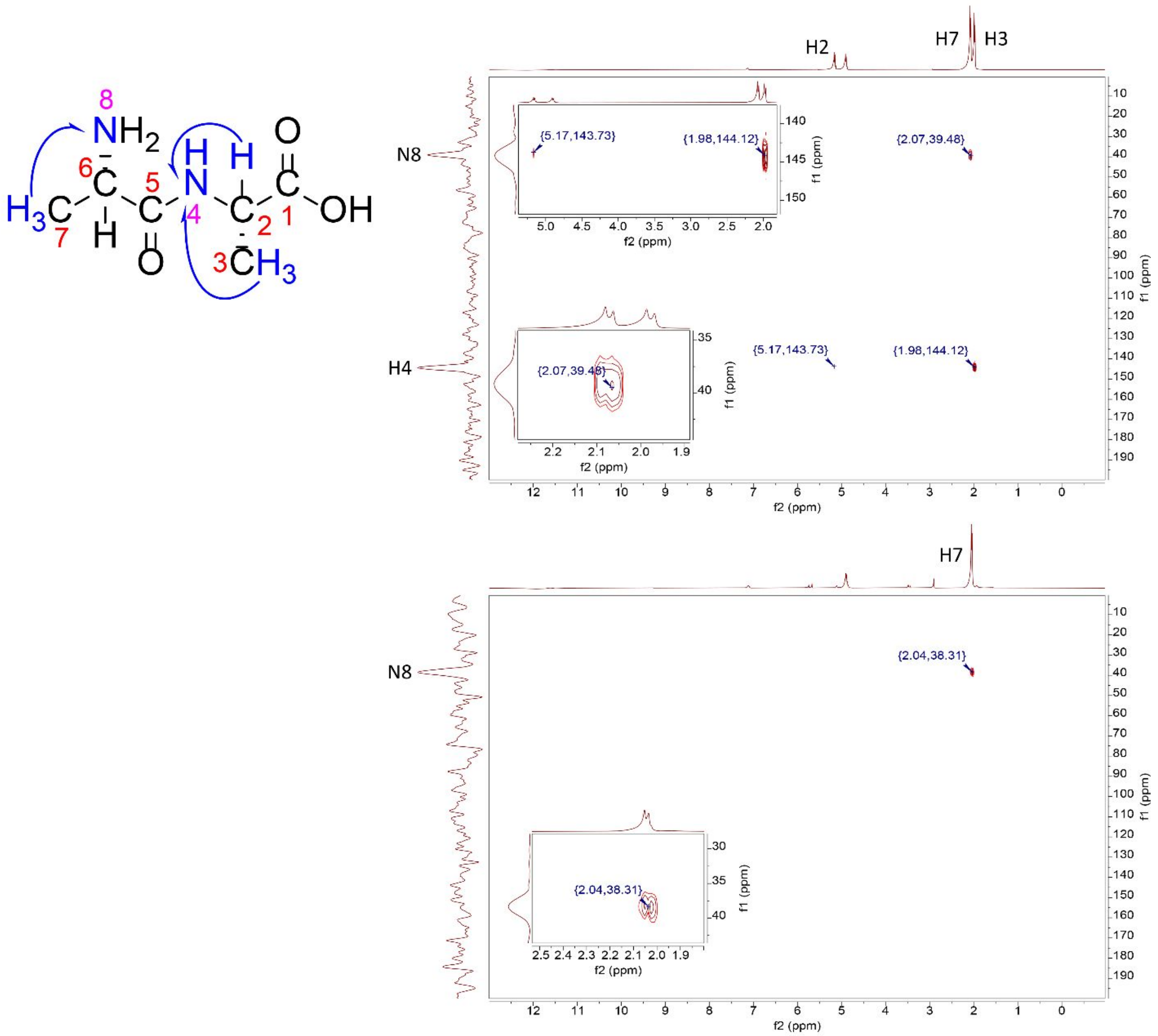

Figure S3. 2D ^{1}H-^{15}N HMBC NMR spectra for AA (**1**) in concentrated sulfuric acid (98% D_2SO_4 and 2% D_2O, by weight) at room temperature after 3 day long incubation (top panel) and 2 month long incubation (bottom panel). The solvent signal is suppressed for clarity. Top panel: The 2D ^{1}H-^{15}N HMBC NMR shows ^{1}H to ^{15}N coupling over 2-3 bonds allowing for the determination of atomic connectivity within a molecule (blue arrows). The top panel spectrum shows three signals at the expected positions. The two signals (5.17 ppm, 143.73 ppm; 1.98 ppm, 144.12 ppm) correspond to a distance of 2 and 3 chemical bonds between H2 and the hydrogen atoms of the methyl group (H3) respectively and the peptide nitrogen N4, confirming the presence of the peptide bond. The signal (2.07 ppm, 39.48 ppm), corresponding to a 3 chemical bonds distance between H7 and N8 indicates the presence of the free amino group. Bottom panel: After 2 month long incubation 98% w/w D_2SO_4 the signal corresponding to the peptide bond amide nitrogen is absent and only free amino group signal remains, confirming the complete solvolysis of the peptide bond.

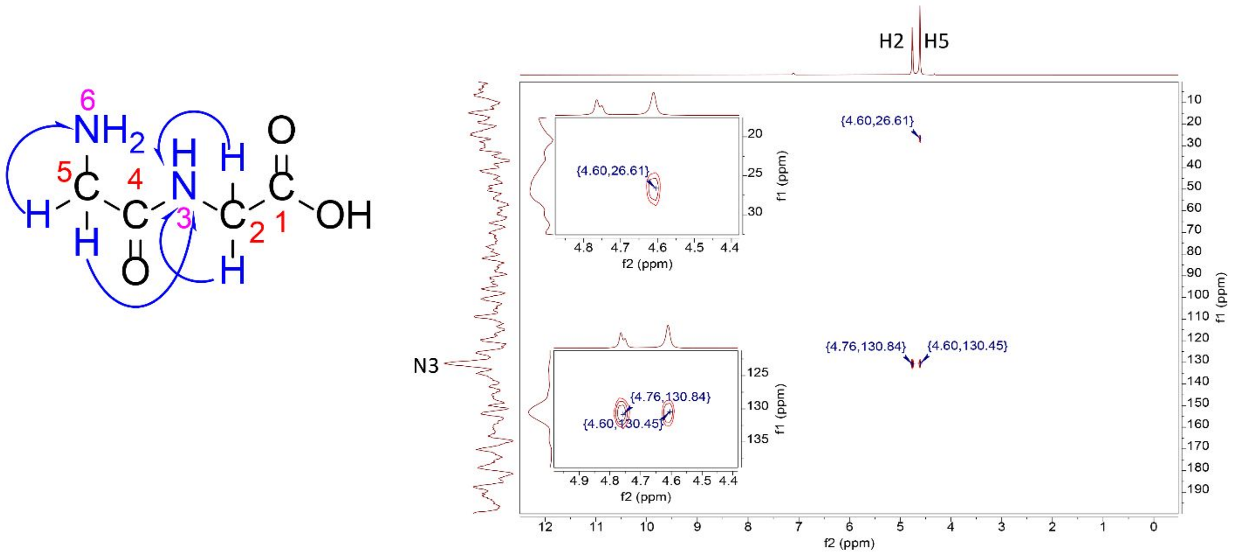

Figure S4. 2D ^{1}H-^{15}N HMBC NMR spectra for GG (**2**) in concentrated sulfuric acid (98% D_2SO_4 and 2% D_2O, by weight) at room temperature after 2 month long incubation. The solvent signal is suppressed for clarity. The 2D ^{1}H-^{15}N HMBC NMR shows ^{1}H to ^{15}N coupling over 2-3 bonds allowing for the determination of atomic connectivity within a molecule (blue arrows). The spectrum shows three signals at the expected positions. The two signals (4.76 ppm, 130.84 ppm; 4.60 ppm, 130.45 ppm) correspond to a distance of 2 and 3 chemical bonds between H2 and H5 respectively and the peptide nitrogen N3, confirming the presence of the peptide bond. The signal (4.60 ppm, 26.61 ppm), corresponding to a 2 chemical bonds distance between H5 and N6 indicates the presence of the free amino group. After 2 month long incubation in the 98% w/w D_2SO_4 the signal corresponding to the peptide bond amide nitrogen is clearly present, confirming the stability of the peptide bond.

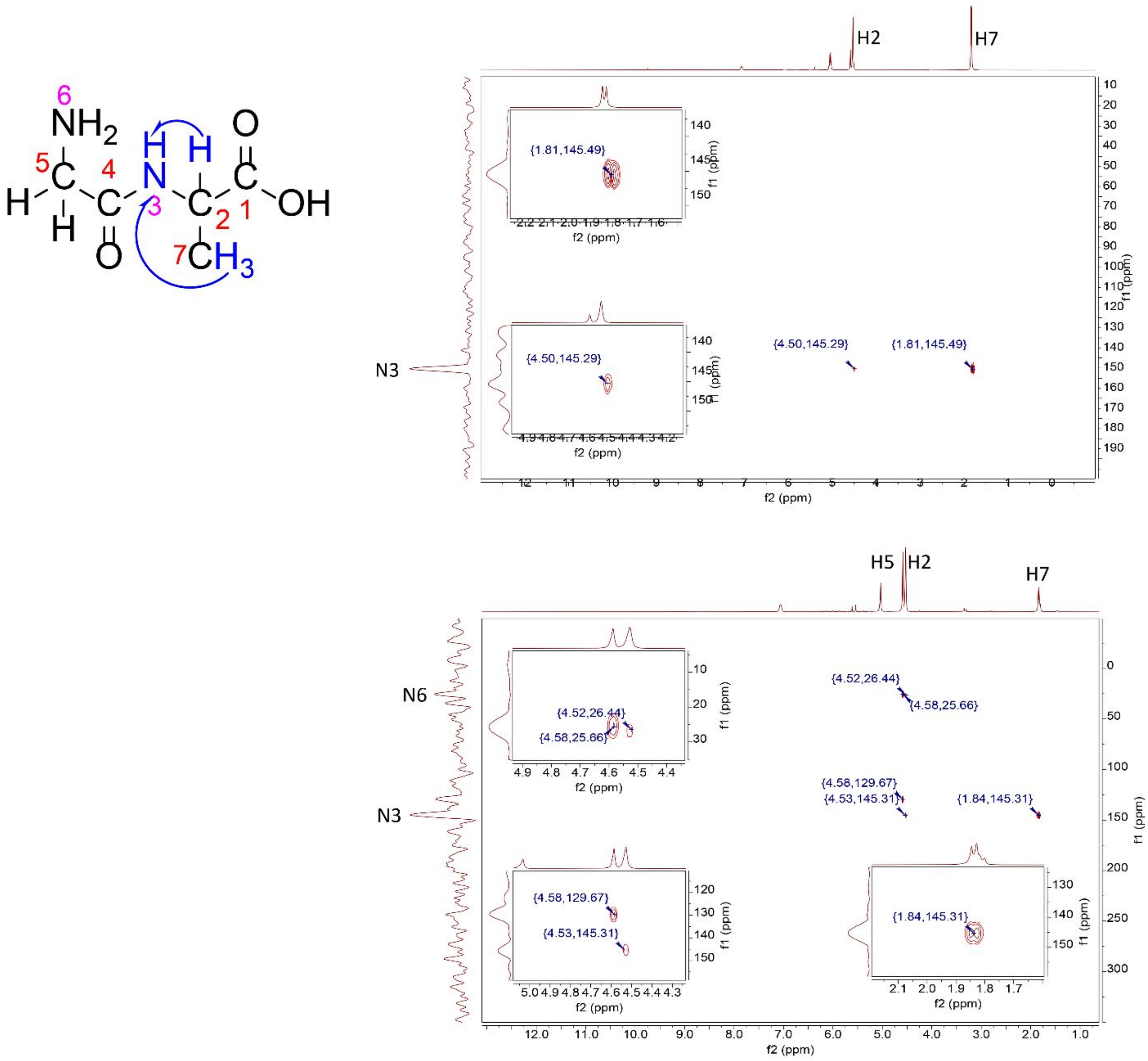

Figure S5. 2D ^1H-^{15}N HMBC NMR spectra for GA (**1**) in concentrated sulfuric acid (98% D_2SO_4 and 2% D_2O, by weight) at room temperature after 3 day long incubation (top panel) and 2 month long incubation (bottom panel). The solvent signal is suppressed for clarity. Top panel: The 2D ^1H-^{15}N HMBC NMR shows ^1H to ^{15}N coupling over 2-3 bonds allowing for the determination of atomic connectivity within a molecule (blue arrows). The top panel spectrum shows two signals at the expected positions. The two signals (4.50 ppm, 145.29 ppm; 1.81 ppm, 145.49 ppm) correspond to a distance of 2 and 3 chemical bonds between H2 and the hydrogen atoms of the methyl group (H7) respectively and the peptide nitrogen N3, confirming the presence of the peptide bond. Bottom panel: After 2 month long incubation 98% w/w D_2SO_4 the free amino group signal becomes predominant. The signal corresponding to the peptide bond amide nitrogen is still present in the solution indicating less efficient solvolysis of the peptide bond in the GA dipeptide than in the case of AA dipeptide.

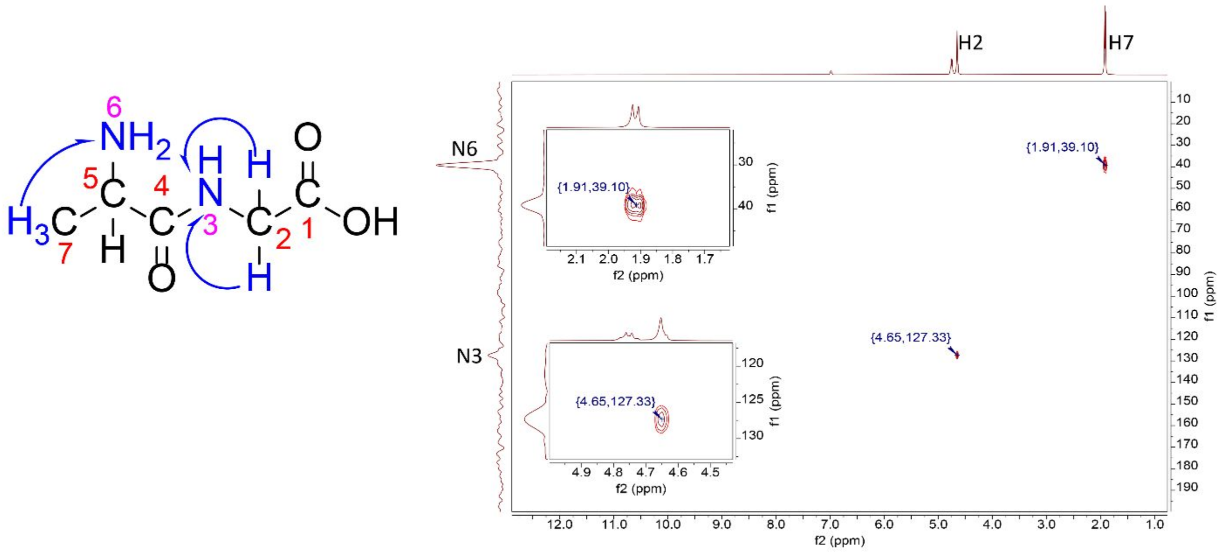

Figure S6. 2D ^{1}H-^{15}N HMBC NMR spectra for AG (**4**) in concentrated sulfuric acid (98% D_2SO_4 and 2% D_2O (by weight) at room temperature after 2 month long incubation. The solvent signal is suppressed for clarity. The 2D ^{1}H-^{15}N HMBC shows ^{1}H to ^{15}N coupling over 2-3 bonds allowing for the determination of atomic connectivity within a molecule (blue arrows). The spectrum shows two signals at the expected positions. The signal (4.65 ppm, 127.33 ppm) correspond to a distance of 2 chemical bonds between H2 and the peptide nitrogen N3, confirming the presence of the peptide bond. The signal (1.91 ppm, 39.10 ppm), corresponding to a 3 chemical bonds distance between hydrogen atoms of the methyl group (H7) and N6 indicates the presence of the free amino group. After 2 month long incubation in the 98% w/w D_2SO_4 the signal corresponding to the peptide bond amide nitrogen (4.65 ppm, 127.33 ppm) is clearly present, confirming the stability of the peptide bond.

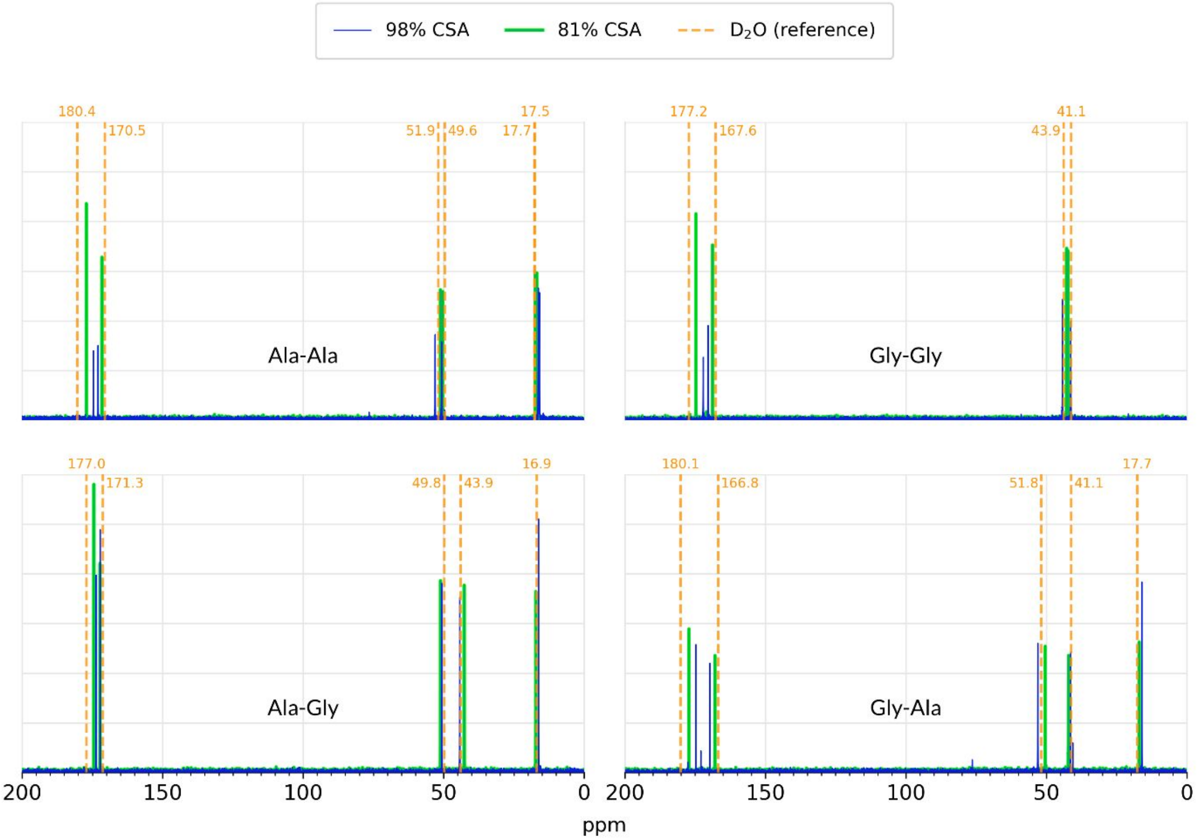

Figure S7. ^{13}C NMR spectra of the four tested dipeptides (**1-4**) in 98% w/w (blue spectra) and 81% w/w (green spectra) concentrated sulfuric acid (CSA) collected after 1 day incubation compared to the literature ^{13}C NMR spectra collected in D_2O[1] (orange dashed line). Each panel shows the ^{13}C NMR spectral peak intensities as a function of shifts in ppm for all four dipeptides (as labeled) after 1 day in concentrated sulfuric acid at room temperature. We show ^{13}C NMR spectral peak shifts for the same dipeptides in D_2O (orange dashed line) from literature values[1]. The spectral peak shifts for 98% w/w and 81% w/w sulfuric acid largely agree with the chemical shift values in D_2O.

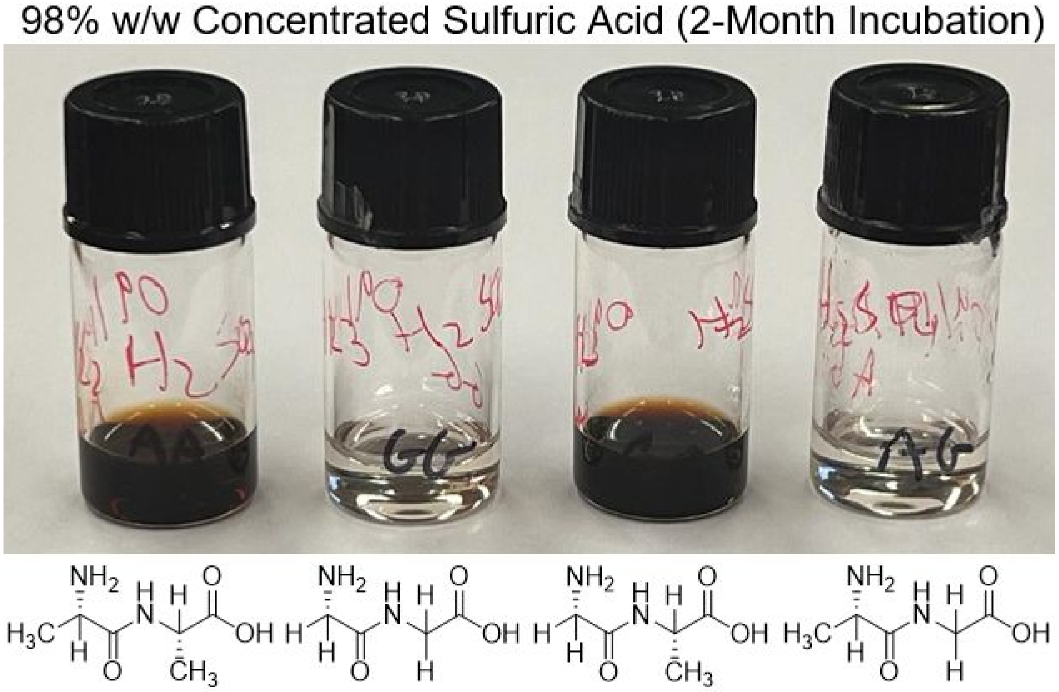

alanylalanine; AA (**1**) glycylglycine; GG (**2**) glycylalanine; GA (**3**) alanylglycine; AG (**4**)

Figure S8. 2-month long incubation of equal amounts of tested dipeptides in 98% w/w sulfuric acid. The solutions of AA and GA in 98% w/w sulfuric acid turn yellow immediately and a very dark red to brown after a couple of months, while GG and AG remain clear and unchanged over the duration of the experiment. The dark red color results from byproducts of the reactions, colloquially called “red oil”, between various organic molecules in concentrated sulfuric acid[19–21].

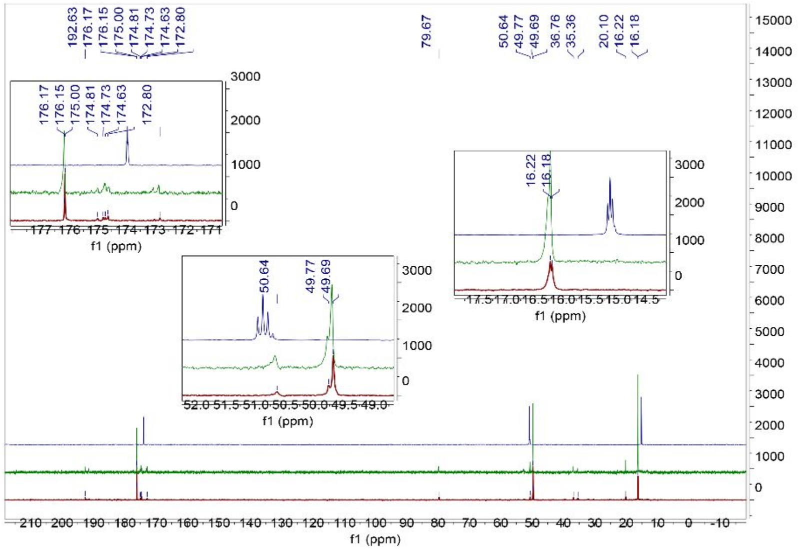

Figure S9. Comparison of the ^{13}C NMR of AA collected in concentrated sulfuric acid (98% D_2SO_4 and 2% D_2O, by weight), at room temperature after 2-month incubation (green spectra) and 4 month incubation (red spectra) to single amino acid alanine (purple spectra) [22]. The 4-month incubation of AA does not lead to further changes of the reaction products. Long-term incubation results suggest that the dominant products are stable to further reactivity in 98% w/w concentrated sulfuric acid.

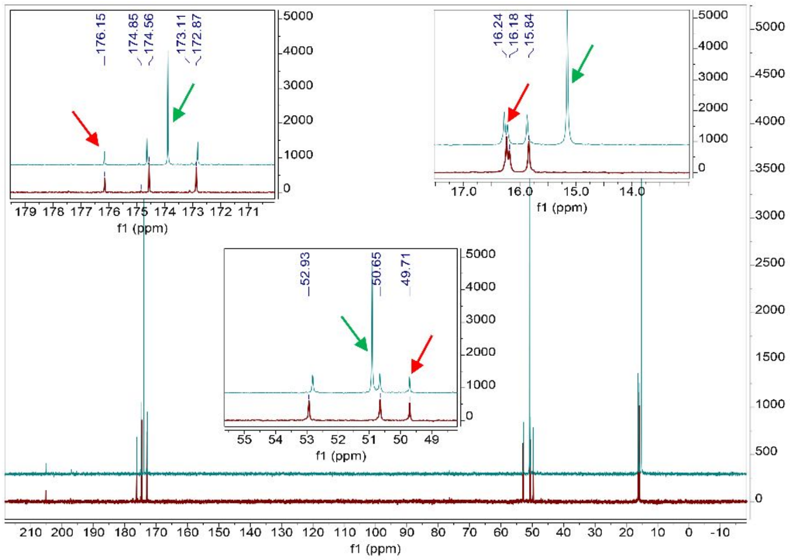

Figure S10. Comparison of the ^{13}C NMR of AA collected after 7 day long incubation [22] in concentrated sulfuric acid (98% D_2SO_4 and 2% D_2O, by weight), at room temperature (red spectra) to the spectra of the same AA sample spiked with single amino acid alanine (teal spectra). The spectra comparison shows that peaks corresponding to the spiked L-Ala amino acid (green arrow) do not overlap with the peaks of the emerging dominant solvolysis product (red arrow). This result confirms that single, unmodified alanine is not the product of the solvolysis of the AA dipeptide in 98% w/w sulfuric acid.

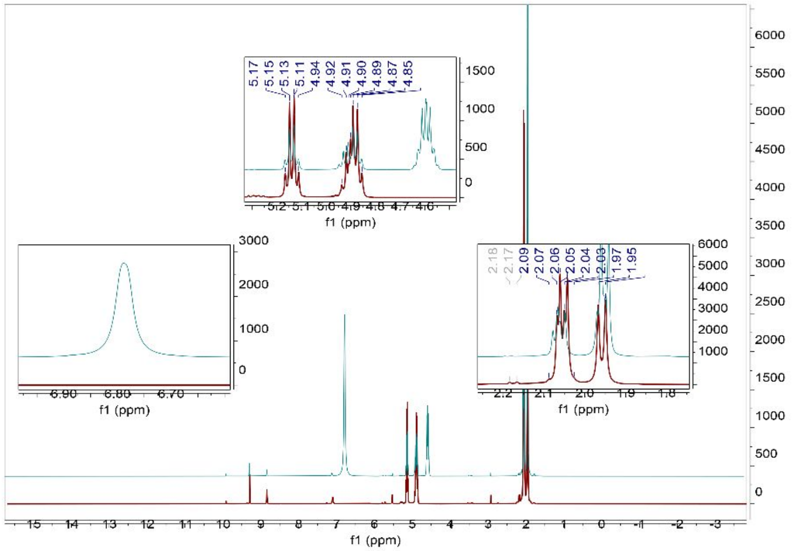

Figure S11. Comparison of the ^{1}H NMR of AA collected after 7 day long incubation [22] in concentrated sulfuric acid (98% D_2SO_4 and 2% D_2O, by weight), at room temperature (red spectra) to the spectra of the same AA sample spiked with single amino acid alanine (teal spectra). The spectra comparison shows that the spectra of the spiked L-Ala amino acid do not overlap with the spectra of the solvolysis products. This result confirms that single, unmodified alanine is not the product of the solvolysis of the AA dipeptide in 98% w/w sulfuric acid.

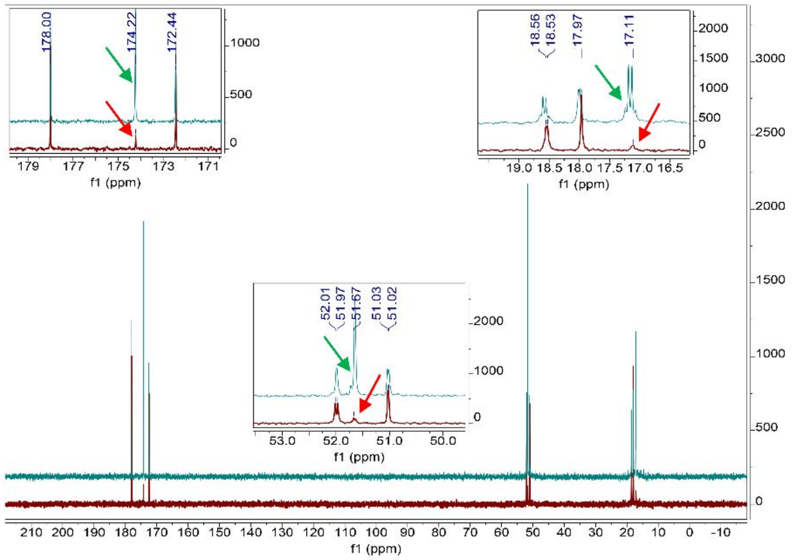

Figure S12. Comparison of the ^{13}C NMR of AA collected after 7 day long incubation [22] in concentrated sulfuric acid (81% D_2SO_4 and 19% D_2O, by weight), at room temperature (red spectra) to the spectra of the same AA sample spiked with single amino acid alanine (teal spectra). The spectra comparison shows that peaks corresponding to the spiked L-Ala amino acid (green arrow) overlap with the peaks of the emerging hydrolysis product (red arrow). This result confirms that, as expected, single, unmodified alanine is the product of the hydrolysis of the AA dipeptide in 81% w/w sulfuric acid.

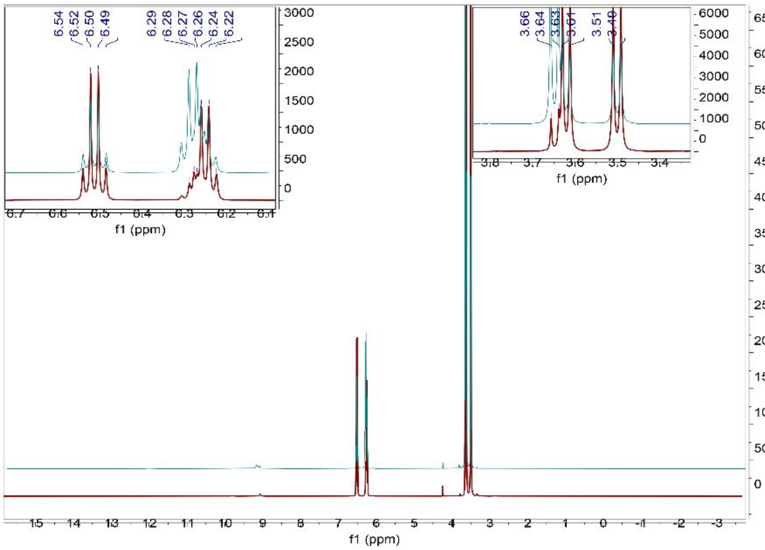

Figure S13. Comparison of the ^{1}H NMR of AA collected after one-week long incubation [22] in concentrated sulfuric acid (81% D_2SO_4 and 19% D_2O, by weight), at room temperature (red spectra) to the spectra of the same AA sample spiked with single amino acid alanine (teal spectra). The spectra comparison shows that the spectra of the spiked L-Ala amino acid overlap with the spectra of the hydrolysis products. This result confirms that single, unmodified alanine is the product of the solvolysis of the AA dipeptide in 81% w/w sulfuric acid.

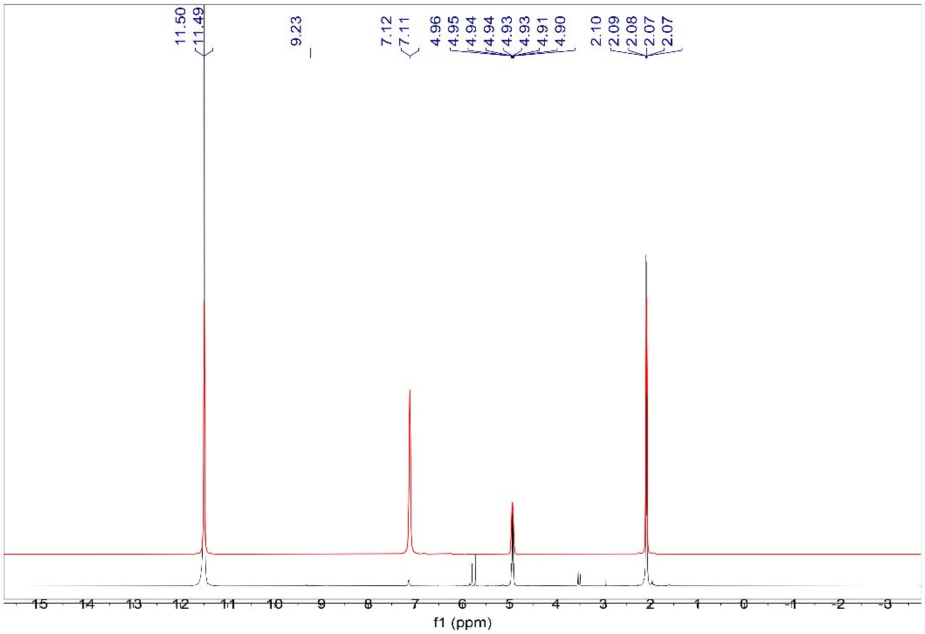

Figure S14. Comparison of the ^{1}H NMR of AA collected after 4-month long incubation [22] in concentrated sulfuric acid (98% D_2SO_4 and 2% D_2O, by weight), at room temperature (black spectra) to the spectra of alaninamide (red spectra). The comparison shows that the spectra of alaninamide overlap with the spectra of the solvolysis products. This result confirms that alaninamide is the product of the solvolysis of the AA dipeptide in 98% w/w sulfuric acid.

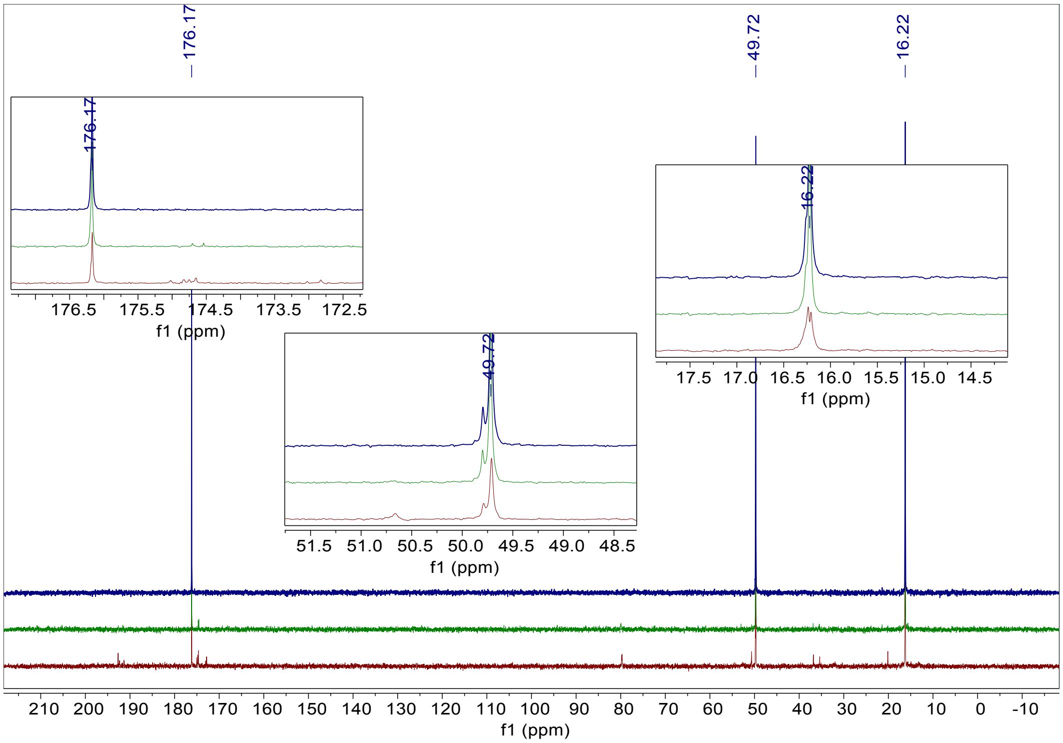

Figure S15. Comparison of the ^{13}C NMR of AA collected after 4 month long incubation [22] in concentrated sulfuric acid (98% D_2SO_4 and 2% D_2O, by weight), at room temperature (red spectra) to the spectra of the AA sample spiked with alaninamide (green spectra) and to the alaninamide alone (blue spectra). The spectra comparison shows that peaks corresponding to the spiked alaninamide overlap with the peaks of the emerging dominant solvolysis product. This result further confirms that alaninamide is the product of the solvolysis of the AA dipeptide in 98% w/w sulfuric acid.

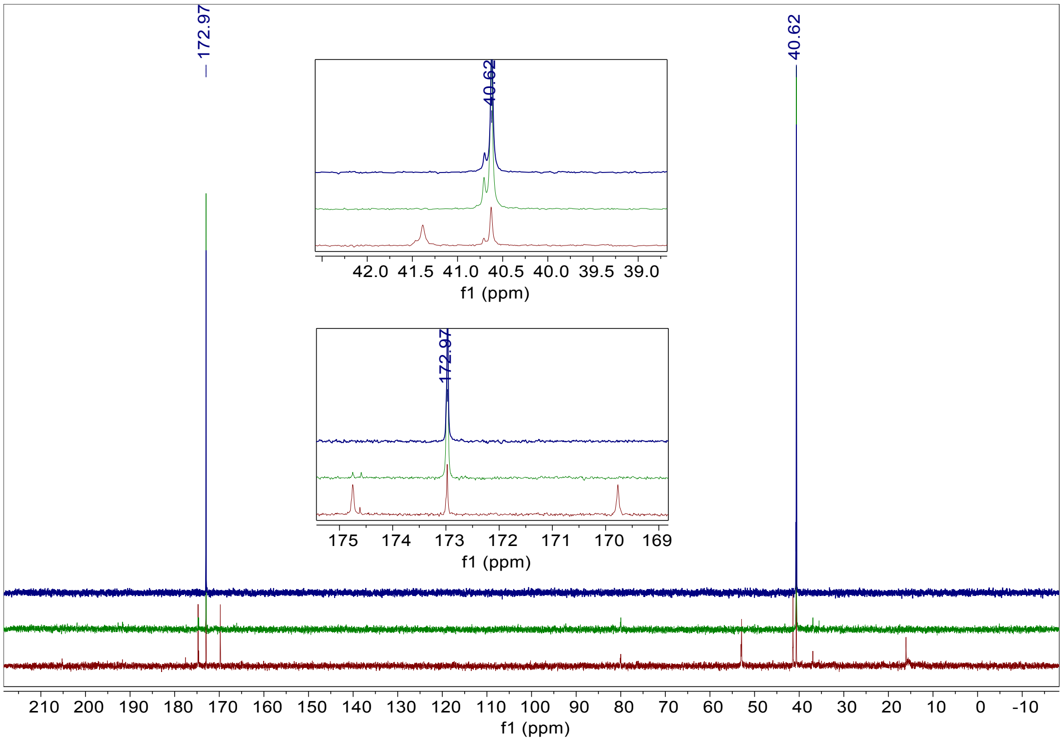

Figure S16. Comparison of the ^{13}C NMR of GA collected after 1 month long incubation in concentrated sulfuric acid (98% D_2SO_4 and 2% D_2O, by weight), at room temperature (red spectra) to the spectra of the GA sample spiked with glycinamide (green spectra) and to the glycinamide alone (blue spectra). The spectra comparison shows that peaks corresponding to the spiked glycinamide overlap with the peaks of the emerging dominant solvolysis product. This result further confirms that glycinamide is the product of the solvolysis of the GA dipeptide in 98% w/w sulfuric acid.

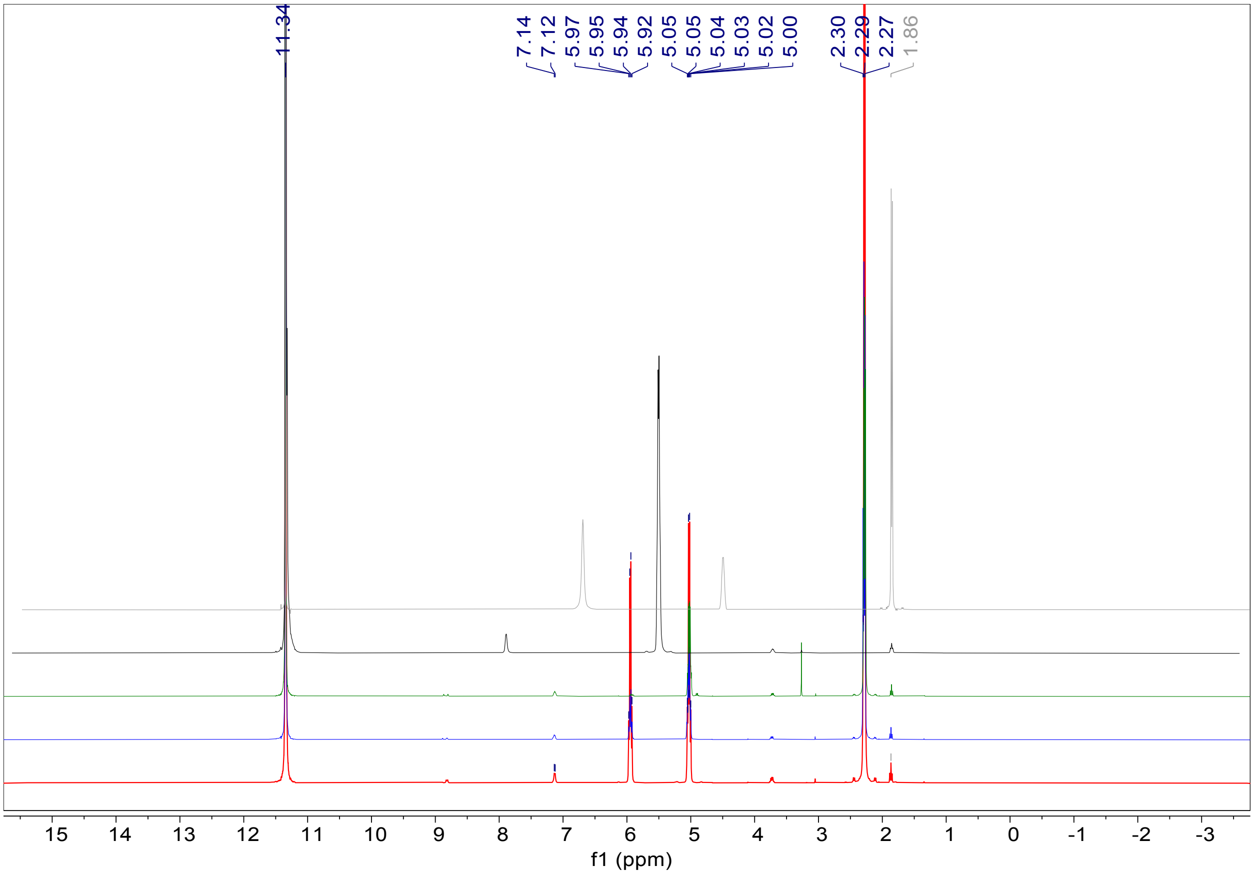

Figure S17. ^{1}H NMR spectra of A3FA in concentrated sulfuric acid (98% D_2SO_4 and 2% D_2O, by weight), at room temperature, collected after 1 day incubation (red spectra), 7 day incubation (blue spectra) to spectra collected after 1 month incubation (green spectra) and compared to single amino acid, trifluoroalanine (black spectra) or alanine (gray spectra). We do not see any significant degradation of the A3FA dipeptide in 98% w/w D_2SO_4 for up to 1 month.

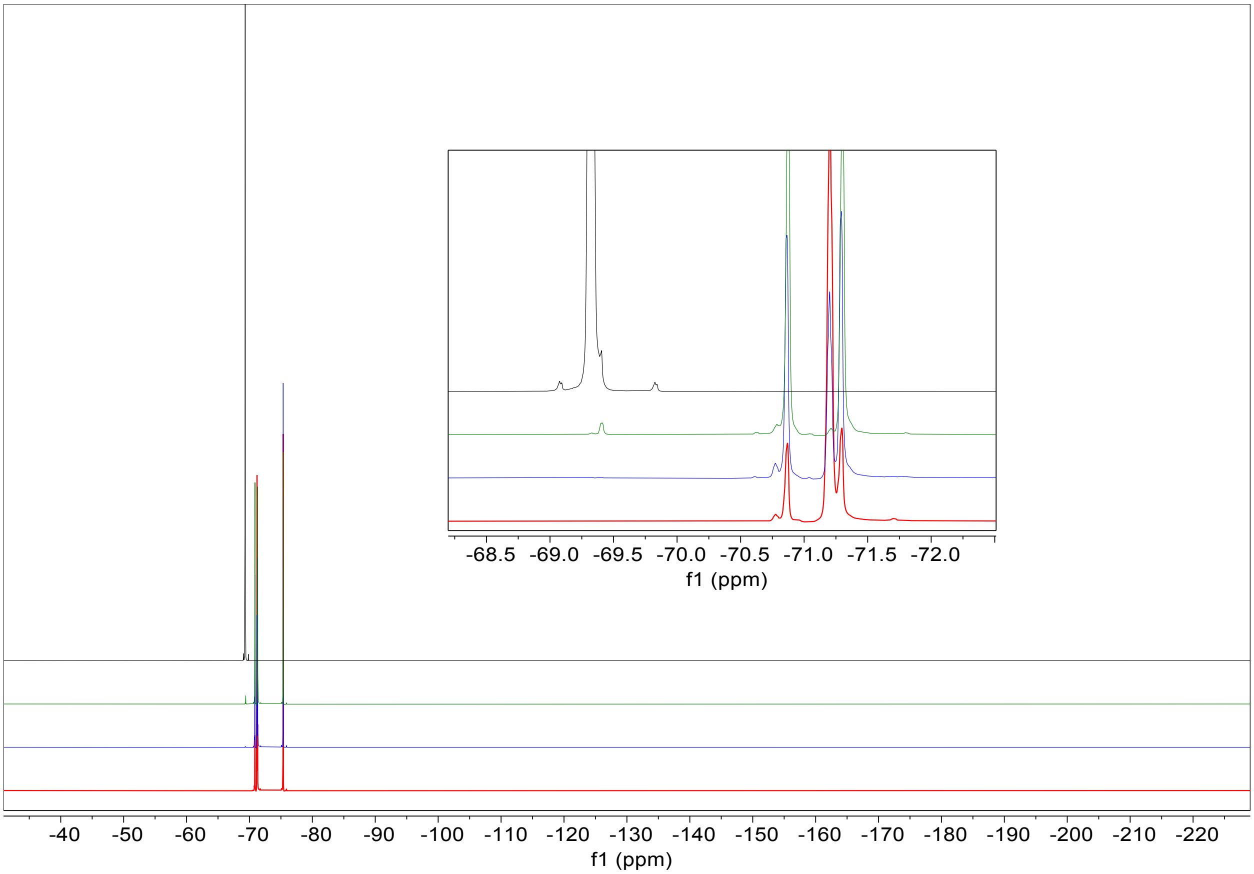

Figure S18. ^{19}F NMR spectra of A3FA dipeptide (**5**) in concentrated sulfuric acid (98% D_2SO_4 and 2% D_2O, by weight), at room temperature, collected after 1 day incubation (red spectra), 7 day incubation (blue spectra) to spectra collected after 1 month incubation (green spectra) and compared to single amino acid, trifluoroalanine (black spectra). We do not see any significant degradation of the A3FA dipeptide in 98% w/w D_2SO_4. Note that the additional peak around 75 ppm comes from the contaminant trifluoroacetic acid (TFA) that is used during the synthesis procedure.

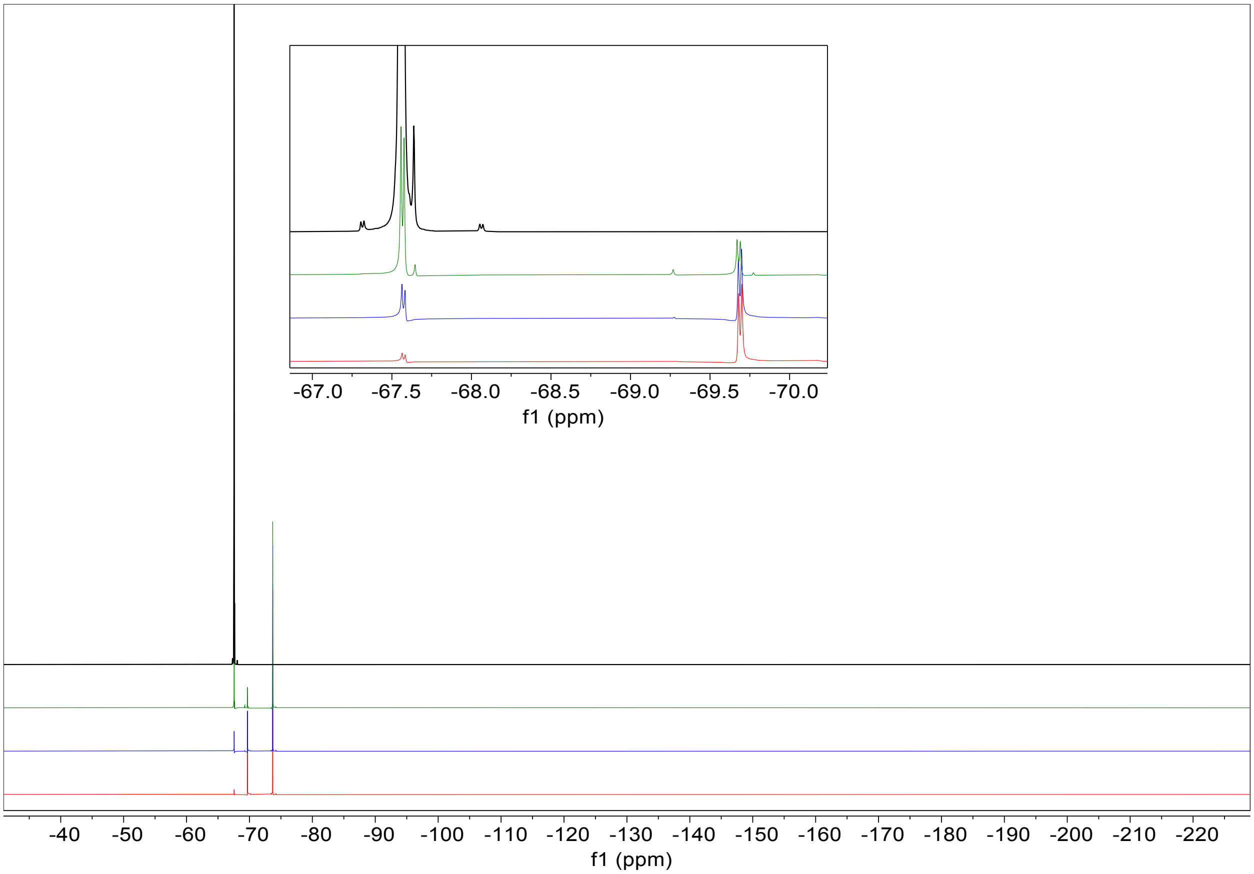

Figure S19. ^{19}F NMR spectra of A3FA (**5**) dipeptide in concentrated sulfuric acid (81% D_2SO_4 and 19% D_2O, by weight), at room temperature, collected after 1 day incubation (red spectra), 7 day incubation (blue spectra) to spectra collected after 1 month incubation (green spectra) and compared to single amino acid, trifluoroalanine (black spectra). The A3FA dipeptide undergoes acid-catalyzed hydrolysis in 81% D_2SO_4 with the release of single amino acid trifluoroalanine. Note that the additional peak around 73 ppm comes from the contaminant trifluoroacetic acid (TFA) that is used during the synthesis procedure.

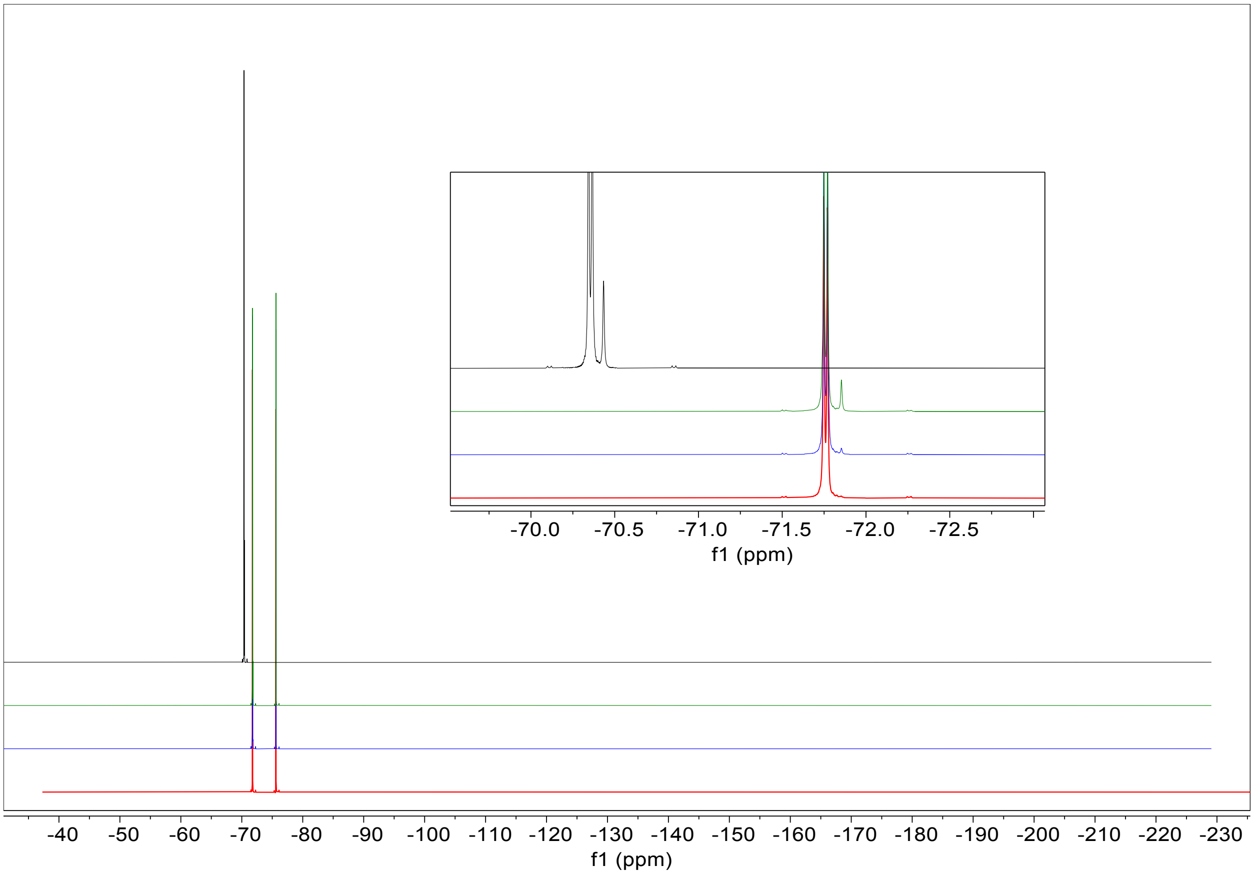

Figure S20. ^{19}F NMR spectra of A3FA (**5**) dipeptide in D_2O, at room temperature, collected after 1 day incubation (red spectra), 7 day incubation (blue spectra) to spectra collected after 1 month incubation (green spectra) and compared to single amino acid, trifluoroalanine (black spectra). Note that the additional peak around 75 ppm comes from the contaminant trifluoroacetic acid (TFA) that is used during the synthesis procedure.

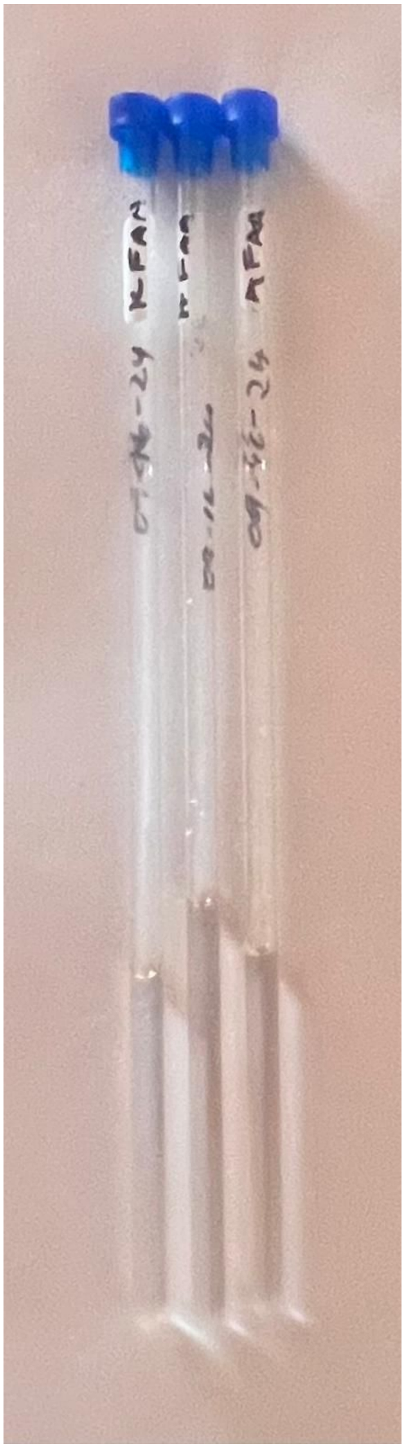

Figure S21. 1-month long incubation of equal amounts of A3FA (**5**) dipeptide in 98% w/w sulfuric acid (left), 81% w/w sulfuric acid (center) and D_2O (right). The three solutions A3FA (**5**) dipeptide remain clear and unchanged over the duration of the experiment. The clear solution of 98% w/w sulfuric acid (left) supports the stability of A3FA (**5**) dipeptide in 98% w/w sulfuric acid over the span of 1 month. The A3FA (**5**) dipeptide is poorly soluble in 81% w/w sulfuric acid (center) which prevents the collection of high quality NMR spectra. We do however expect the solution to be clear in 81% w/w sulfuric acid as well as the acid catalyzed hydrolysis of all the tested dipeptides leads to the release of single amino acids. The individual amino acids (alanine and trifluoroalanine) are stable in concentrated sulfuric acid and have clear solutions. See Supplementary Dataset 5 for the original NMR data of the A3FA (**5**) dipeptide and trifluoroalanine in concentrated sulfuric acid and D_2O.

Supplementary Tables.

AA						
**Solvent (reference)**	**C1 (ppm)**	**C2 (ppm)**	**C3 (ppm)**	**C5 (ppm)**	**C6 (ppm)**	**C7 (ppm)**
D_2O	180.4	51.9	17.7	170.5	49.6	17.5
D_2SO_4 81% (w/w)	177.2	51.2	17.5	171.6	50.3	16.9
D_2SO_4 98% (w/w)	174.6	52.9	16.3	172.9	50.7	15.9

Table S1. Comparison of the ^{13}C NMR chemical shifts of the tested dipeptide obtained after 1 day incubation in concentrated D_2SO_4 to chemical shift values in D_2O reported in the literature [1]. The assignment of the carbon atoms in the AA dipeptide is analogous to the carbon atom assignments from [23]. The numbering of atoms follows the convention shown on Figure S3. ^{13}C NMR chemical shifts are rounded to one digit after the decimal point.

GG				
**Solvent (reference)**	**C1 (ppm)**	**C2 (ppm)**	**C4 (ppm)**	**C5 (ppm)**
D_2O	177.2	43.9	167.6	41.1
D_2SO_4 81% (w/w)	174.7	42.8	168.8	42.3
D_2SO_4 98% (w/w)	172.0	44.2	170.3	41.3

Table S2. Comparison of the ^{13}C NMR chemical shifts of the tested dipeptide obtained after 1 day incubation in concentrated D_2SO_4 to chemical shift values in D_2O reported in the literature [1]. The assignment of the carbon atoms in the GG dipeptide is analogous to the carbon atom assignments from [24]. The numbering of atoms follows the convention shown on Figure S4. ^{13}C NMR chemical shifts are rounded to one digit after the decimal point.

GA					
**Solvent (reference)**	**C1 (ppm)**	**C2 (ppm)**	**C4 (ppm)**	**C5 (ppm)**	**C7 (ppm)**
D_2O	180.1	51.8	166.8	41.1	17.7
D_2SO_4 81% (w/w)	177.1	50.4	167.9	42.1	17.0
D_2SO_4 98% (w/w)	174.7	53.0	169.7	41.3	16.0

Table S3. Comparison of the ^{13}C NMR chemical shifts of the tested dipeptide obtained after 1 day incubation in concentrated D_2SO_4 to chemical shift values in D_2O reported in the literature [1]. The numbering of atoms follows the convention shown on Figure S5. ^{13}C NMR chemical shifts are rounded to one digit after the decimal point.

AG					
**Solvent (reference)**	**C1 (ppm)**	**C2 (ppm)**	**C4 (ppm)**	**C5 (ppm)**	**C7 (ppm)**
D_2O	177.0	49.8	171.3	43.9	16.9
D_2SO_4 81% (w/w)	174.4	51.1	172.2	42.7	17.2
D_2SO_4 98% (w/w)	173.6	50.6	172.2	44.3	16.2

Table S4. Comparison of the ^{13}C NMR chemical shifts of the tested dipeptide obtained after 1 day incubation in concentrated D_2SO_4 to chemical shift values in D_2O reported in the literature [1]. The numbering of atoms follows the convention shown on Figure S6. ^{13}C NMR chemical shifts are rounded to one digit after the decimal point.

A3FA						
**Solvent (reference)**	**C1 (ppm)**	**C2 (ppm)**	**C3 (ppm)**	**C5 (ppm)**	**C6 (ppm)**	**C7 (ppm)**
D_2O	167.7	54.6*	*122.8	171.1	49.1	16.4
D_2SO_4 81% (w/w)	—**	—**	—**	—**	—**	—**
D_2SO_4 98% (w/w)	167.3	55.4*	121.6*	172.8	51.6	16.8

Table S5. Comparison of the ^{13}C NMR chemical shifts of the tested dipeptide obtained after 1 day incubation in concentrated D_2SO_4 to chemical shift values in D_2O. The numbering of atoms follows the convention shown on Figure S3. ^{13}C NMR chemical shifts are rounded to one digit after the decimal point. *The C2 and C3 carbon peaks get split into quadruplets. ** The spectra could not be collected due to poor solubility of A3FA dipeptide in 81% w/w sulfuric acid.

Dominant product of solvolysis	*Pros*	*Cons*
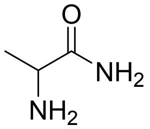 2-aminopropanamide	1. Matches the number of carbon peaks of the dominant product on the ^{13}C NMR.<br>2. The ^{13}C NMR chemical shifts of the dominant product carbon peaks are consistent with the C-term. modified alanine.<br>3. The ^{13}C (Figures 3, S15-S16) and ^{1}H NMR (Figures S14) spectra of alaninamide (2-aminopropanamide) match the spectra of the dominant product of the solvolysis.	
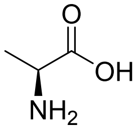 *L*-alanine	1. The expected product of the acid-catalyzed hydrolysis of the peptide bond.	1. The NMR spectra of the dominant product does not match the spectra of single L-Ala amino acid (Figure 1). Conclusively ruled out by the “spiking experiments” (Figures S10, S11).
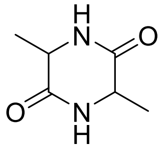 3,6-dimethylpiperazine-2,5-dione	1. Concentrated sulfuric acid is a dehydrating agent. Dehydration reaction could lead to the cyclization of the Ala-Ala dipeptide.<br>2. The molecule is symmetric matching the number of carbon peaks of the dominant product on the ^{13}C NMR.	1. 2D ^{1}H-^{15}N NMR spectra shows no distinct amide signal (Figure S3) [7].<br>2. The cyclization of the dipeptide via the dehydration reaction should proceed for all four dipeptides. GG and AG dipeptides remain unchanged.<br>3. If cyclization is the only reactivity of the AA dipeptide then no other products besides the cyclic dipeptide should emerge. We do however see diverse minor products of the solvolysis reaction.
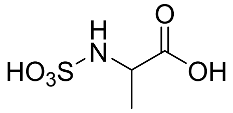 *N*-sulfoalanine	1. Matches the number of carbon peaks of the dominant product on the ^{13}C NMR.	1. ^{1}H-^{15}N HMBC NMR spectra do not support the N-sulfation of the α-amino group (Figure S3).
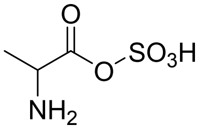 2-aminopropanoic sulfuric anhydride	1. Matches the number of carbon peaks of the dominant product on the ^{13}C NMR.<br>2. The ^{13}C NMR chemical shifts of the dominant product carbon peaks are consistent with the C-term. modified alanine.<br>3. Formation of carboxylic sulfuric anhydrides occurs in concentrated sulfuric acid (e.g. [11]).	1. No evidence for sulfation of carboxylic group of amino acids.

Table S6. An overview of possible dominant products of the solvolysis of the AA dipeptide in 98% w/w sulfuric acid and the data that support (pros) or do not support them (cons).

Supplementary References:

(1) Furukawa, S.; Fukuyama, T.; Matsui, A.; Kuratsu, M.; Nakaya, R.; Ineyama, T.; Ueda, H.; Ryu, I. Coupling-Reagent-Free Synthesis of Dipeptides and Tripeptides Using Amino Acid Ionic Liquids. *Chem. Eur. J.* **2015**, *21* (34), 11980–11983.

(2) Seager, M. D.; Seager, S.; Bains, W.; Petkowski, J. J. Stability of 20 Biogenic Amino Acids in Concentrated Sulfuric Acid: Implications for the Habitability of Venus' Clouds. *Astrobiology* **2024**, *24* (4), 386–396.

(3) Seager, S.; Petkowski, J. J.; Seager, M. D.; Grimes Jr, J. H.; Zinsli, Z.; Vollmer-Snarr, H. R.; Abd El-Rahman, M. K.; Wishart, D. S.; Lee, B. L.; Gautam, V.; Herrington, L.; Bains, W.; Darrow, C. Stability of Nucleic Acid Bases in Concentrated Sulfuric Acid: Implications for the Habitability of Venus' Clouds. *Proc. Natl. Acad. Sci.* **2023**, *120* (25), e2220007120. https://doi.org/10.1073/pnas.2220007120.

(4) Seager, S.; Petkowski, J. J.; Seager, M. D.; Grimes Jr, J. H.; Zinsli, Z.; Vollmer-Snarr, H. R.; Abd El-Rahman, M. K.; Wishart, D. S.; Lee, B. L.; Gautam, V.; Herrington, L.; Bains, W.; Darrow, C. Year-Long Stability of Nucleic Acid Bases in Concentrated Sulfuric Acid: Implications for the Persistence of Organic Chemistry in Venus' Clouds. *Life* **2024**, *14*, 538. https://doi.org/10.3390/life14050538.

(5) Lorenzi, G. P.; Rizzo, V.; Thoresen, F.; Tomasic, L. Circular Dichrosim and Conformational Equilibrium of Homopoly-L-Peptides with Alkyl Side Chains in Concentrated Sulfuric Acid. *Macromolecules* **1979**, *12* (5), 870–874.

(6) Steigman, J.; Peggion, E.; Cosani, A. Protonation of Peptides. I. Behavior of a Model Diamide and of Poly (. Gamma.-Ethyl-L-Glutamate) in Strong Acid-Water Mixtures. *J. Am. Chem. Soc.* **1969**, *91* (7), 1822–1829.

(7) Kricheldorf, H. R. 15N NMR Spectroscopy. 19—Spectroscopic Characterization of Cyclodipeptides (2, 5-dioxopiperazines). *Org. Magn. Reson.* **1980**, *13* (1), 52–58.

(8) Lustig, B.; Kondritzer, A. A. The Effect of Concentrated Acid on Hair and Wool. *Arch. Biochem.* **1945**, *8* (1), 51–56.

(9) Harris, M.; Mease, R.; Rutherford, H. The Reaction of Wool with Strong Solutions of Sulfuric Add. *J. Res. Natl. Bur. Stand* **1937**, *18*, 343–350.

(10) Reitz, H. C.; Ferrel, R. E.; Fraenkel-Conrat, H.; Olcott, H. S. Action of Sulfating Agents on Proteins and Model Substances. I. Concentrated Sulfuric Acid. *J. Am. Chem. Soc.* **1946**, *68* (6), 1024–1031.

(11) Campaigne, E.; Suter, C. M. The Action of Sulfuric Acid on Tertiary Carboxylic Acids. *J. Am. Chem. Soc.* **1946**, *68* (5), 880–882.

(12) Gillespie, R. J.; Leisten, J. A. The Behaviour of Organic Compounds in Sulphuric Acid. *Q. Rev. Chem. Soc.* **1954**, *8* (1), 40–66.

(13) Gillespie, R. J. 585. Cryoscopic Measurements in Sulphuric Acid. Part VIII. The Solutes Acetic Anhydride and Benzoic Anhydride. Cryoscopic Evidence for the Acetylium and Benzoylium Ions. *J. Chem. Soc.* **1950**, 2997–3000.

(14) Murray Jr, T. F.; Kenyon, W. O. The Rates of Formation of Sulfoaliphatic Acids. *J. Am.*

Chem. Soc. **1940**, *62* (5), 1230–1233.

(15) Smith, C. J.; Huff, A. K.; Ward, R. M.; Leopold, K. R. Carboxylic Sulfuric Anhydrides. *J. Phys. Chem. A* **2019**, *124* (4), 601–612.

(16) Zhang, H.; Gao, R.; Li, H.; Li, Y.; Xu, Y.; Chai, F. Formation Mechanism of Typical Aromatic Sulfuric Anhydrides and Their Potential Role in Atmospheric Nucleation Process. *J. Environ. Sci.* **2023**, *123*, 54–64.

(17) Zhang, H.; Wang, W.; Li, H.; Gao, R.; Xu, Y. A Theoretical Study on the Formation Mechanism of Carboxylic Sulfuric Anhydride and Its Potential Role in New Particle Formation. *RSC Adv.* **2022**, *12* (9), 5501–5508.

(18) Willcott, M. R. MestRe Nova. *J. Am. Chem. Soc.* **2009**, *131* (36), 13180. https://doi.org/10.1021/ja906709t.

(19) Miron, S.; Lee, R. J. Molecular Structure of Conjunct Polymers. *J. Chem. Eng. Data* **1963**, *8* (1), 150–160.

(20) Albright, L. F.; Houle, L.; Sumutka, A. M.; Eckert, R. E. Alkylation of Isobutane with Butenes: Effect of Sulfuric Acid Compositions. *Ind. Eng. Chem. Process Des. Dev.* **1972**, *11* (3), 446–450.

(21) Huang, Q.; Zhao, G.; Zhang, S.; Yang, F. Improved Catalytic Lifetime of H2SO4 for Isobutane Alkylation with Trace Amount of Ionic Liquids Buffer. *Ind. Eng. Chem. Res.* **2015**, *54* (5), 1464–1469.

(22) Petkowski, J. J.; Seager, M. D.; Bains, W.; Seager, S. General Instability of Dipeptides in Concentrated Sulfuric Acid as Relevant for the Venus Cloud Habitability. *Sci. Rep.* **2024**, *14* (1), 17083.

(23) Bouř, P.; Buděšínský, M.; Špirko, V.; Kapitán, J.; Šebestík, J.; Sychrovský, V. A Complete Set of NMR Chemical Shifts and Spin− Spin Coupling Constants for L-Alanyl-l-Alanine Zwitterion and Analysis of Its Conformational Behavior. *J. Am. Chem. Soc.* **2005**, *127* (48), 17079–17089.

(24) Saito, T.; Hayamizu, K.; Yanagisawa, M.; Yamamoto, O.; Wasada, N.; Someno, K.; Kinugasa, S.; Tanabe, K.; Tamura, T.; Hiraishi, J. Spectral Database for Organic Compounds (Sdbs). *Natl. Inst. Adv. Ind. Sci. Technol.* **2006**.
